# Circular RNA circIGF1R controls cardiac fibroblast proliferation through regulation of carbohydrate metabolism

**DOI:** 10.1038/s41598-025-07167-3

**Published:** 2025-06-27

**Authors:** Arne Schmidt, Kevin Schmidt, Sonja Groß, Dongchao Lu, Ke Xiao, Dimyana Neufeldt, Sarah Cushman, Nele Lehmann, Sabrina Thum, Angelika Pfanne, Annette Just, Andreas Pich, Alexander Heinz, Karsten Hiller, Hannah Jill Hunkler, Wilson Lek Wen Tan, Roger Foo, Christian Bär, Thomas Thum, Mira Jung

**Affiliations:** 1 Institute of Molecular and Translational Therapeutic Strategies, Hannover Medical School, Hannover, Germany; 2 Fraunhofer Institute for Toxicology and Experimental Medicine (ITEM), Hannover, Germany; 3 Institute of Toxicology, Hannover Medical School, Hannover, Germany; 4 Core Facility Proteomics, Institute of Toxicology, Hannover, Germany; 5 Department of Bioinformatics and Biochemistry, Braunschweig Integrated Centre of Systems Biology (BRICS), Technische Universität Braunschweig, Braunschweig, Germany; 6 Institute of Molecular and Cell Biology, A*STAR, Singapore, Singapore; 7 Center for Translational Regenerative Medicine, Hannover Medical School, Hannover, Germany

**Keywords:** Circular RNA, Cardiac fibrosis, Cardiac fibroblast, Cardiac metabolism, Glycolysis, Proliferation, Heart failure, Cell biology, Drug discovery, Molecular biology, Cardiology, Molecular medicine

## Abstract

Excessive fibroblast proliferation and metabolic reprogramming are hallmarks of pathological cardiac remodeling, contributing significantly to impaired cardiac function. This study investigates the role of circular RNAs (circRNAs) in fibroblast metabolic reprogramming, an unexplored area with potential therapeutic implications. Through deep circRNA sequencing of cardiac tissue from heart failure (HF) patients and healthy individuals, we identified circIGF1R (hsa_circ_0005035), which exhibited dysregulation specifically in isolated cardiac fibroblasts derived from failing hearts. Silencing circIGF1R in patient-derived human cardiac fibroblasts (HCFs) led to accelerated proliferation, enhanced glycolytic activity, altered glucose trafficking, and increased glucose import. Conversely, administering recombinant circIGF1R inhibited the accelerated proliferation and enhanced glycolytic activity observed in HCFs from HF patients. Mechanistically, RNA pulldown assays and in silico analyses identified AZGP1 as a potential interaction partner facilitating the glycolysis-inhibitory and anti-proliferative functions of circIGF1R. Our findings identify circIGF1R as a pivotal regulator of fibroblast proliferation via metabolic reprogramming, particularly by glycolysis inhibition. Overexpression of circIGF1R demonstrated significant anti-fibrotic effects in cardiac fibroblasts derived from heart failure patients. These results underscore the therapeutic potential of circIGF1R in attenuating cardiac fibrosis by directly targeting fibroblast metabolism in the context of pathological cardiac remodeling.

**Supplementary Information:**

The online version contains supplementary material available at 10.1038/s41598-025-07167-3.

## Introduction

Cardiovascular diseases are the leading cause of mortality, accounting for 32% of all deaths worldwide in 2019 (World Health Organization). Heart failure (HF) is a clinical syndrome characterized by impaired contractile function of the myocardium, resulting in insufficient distribution of blood throughout the body. The impaired pumping function of the heart originates from its negligible regenerative capacity, prompting excessive activation and proliferation of cardiac fibroblasts to compensate for damaged cardiomyocytes and endothelial cells^1,2^. Excessive cardiac fibrosis results in stiffening of ventricles and diminished contractility^3^. For decades, fibrosis has been considered an irreversible process. However, recent research suggests that targeting the early proliferative stages of fibroblasts holds promise for developing anti-fibrotic therapies across various organs^4–6^.

Metabolic reprogramming is well known in the failing heart, especially in cardiomyocytes^7^. However, there is increasing evidence that altered metabolism is also related to fibroblast activation and fibrotic disease progression^8^. Mitochondrial respiration and β-oxidation of fatty acids is heavily impaired in the failing heart, resulting in a severe energy deficit, which is compensated by switching cardiac metabolism back into a fetal phenotype and an elevated consumption of carbohydrates^7–10^. Elevated glycolysis is linked to various pathological conditions such as HF and cancer, and stimulates the activation of fibroblasts by promoting proliferation, α-SMA expression, and collagen synthesis^11^. Not only does glycolysis enable fibroblasts to rapidly generate adenosine triphosphate (ATP) and lactate to meet increased energy demands, but it also provides substrates in the form of glycolysis intermediates to fuel the aforementioned processe^11–13^. Consequently, inhibiting glycolysis reversed transdifferentiation into myofibroblasts and alleviated fibroblast proliferation in fibrotic disease^11,13^. Likewise, abrogating aerobic glycolysis during cardiac hypertrophy and HF, restored mitochondrial metabolism and improved cardiac function^9^.

Circular RNAs (circRNAs) are a class of non-coding RNAs characterized by their enclosed loop structure, which is formed through a non-canonical splicing event known as backsplicing^14,15^. Circular RNAs exhibit ubiquitous expression across various tissues and are subject to tissue-specific regulatory mechanisms. Moreover, their significant role in (patho)physiological processes highlight them as novel therapeutic targets for various diseases^16,17^. In this context, we recently demonstrated that circ-INSR attenuates doxorubicin-induced cardiotoxicity by sustaining mitochondrial function and improving cardiomyocyte survival^18 ^whereas mitochondrial fission and apoptosis-related circRNA (MFACR) regulates cardiomyocyte autophagy and mitochondrial fission by sponging miR-652-3p^19^. Moreover, silencing of circHelz and circSmad4 alleviated cardiac fibrosis by inhibiting cardiac fibroblast proliferation and migration, as well as attenuating myofibroblast activation^20,21^. However, the specific link between metabolism-associated circRNAs and fibroblast activation in HF remains largely unexplored.

Despite the increasing evidence indicating that circRNAs regulate many biological processes, current evidence on their roles in cardiac disease, particularly related to cardiac fibrosis, remains scarce. In this study, we identified and characterized a novel circRNA that is involved in metabolic state regulation in cardiac fibroblasts from HF patients.

## Materials and methods

### RNA sequencing and in Silico validation

Total RNA of human HF patients^22^ (Supplemental Table 1) and healthy control heart biopsies as well as RNA libraries were prepared and RNA sequencing was conducted as previously described^18^. CircRNA sequences were retrieved from circbase^23^ and sequencing reads were visualized using the integrative genome browser (igv) (version 2)^24^ to identify the backsplice junction (BSJ) of potential circRNA candidates. Homology of circRNA candidates was assessed via the UCSC Genome Browser^25,26^.

### Cell culture

Human cardiac fibroblasts (HCFs) were either purchased from PromoCell (Supplemental Table 2) or isolated from HF patients (Supplemental Table 3) and cultured in FGM-3 (FBM-3 (PromoCell), supplemented with 10% FBS (Gibco), penicillin-streptomycin (100 U/ml and 100 µg/ml, Gibco), 1 ng/ml hbFGF (PromoCell) and 5 µg/ml hINS (PromoCell)). Following expansion, HCFs were aliquoted and frozen in Cryo-SFM (PromoCell) for long-term storage. HCFs were used in passages 6 to 9 for all functional experiments to avoid senescence of cardiac fibroblasts. Further treatments as well as in vitro assays were performed as described in the supplementary material.

### Modulation of circRNA expression

The inhibition of circIGF1R was achieved by transfection of siRNAs specifically targeting the BSJ of circIGF1R (Table 1). For all experiments shown, an equal mixture of three siRNAs was transfected in a total siRNA concentration of 100 nM. Silencer™ Negative Control 1 (#4611, Ambion) was utilized as a negative control siRNA (NC siRNA) and transfection of siRNAs was performed with Lipofectamine™ RNAiMAX (Invitrogen). The transfection mixture was replaced by culture medium after 6 h and cells were harvested 24–72 h following transfection for further experiments.

**Table 1:** BSJ-complementary siRNAs.

siRNA	Sense sequence (5’→3’)
hsa_circIGF1R #1	CGCUGCCAGAAAAUCUGCGG
hsa_circIGF1R #2	AGAAAUCUGCGGGCCAGG
hsa_circIGF1R #3	CCAGAAAAUCUGCGGGCCA

Similarly, overexpression of circIGF1R was conducted by transfection with circIGF1R mimics. CircIGF1R mimics were generated via in-vitro-transcription (IVT) and synthesized as indicated in the supplementary material (Supplemental Tables 4, 5)^27^. For all experiments, HCFs were transfected with 250 ng/ml circIGF1R mimics, packaged into Lipofectamine™ RNAiMAX. The transfection mixture was replaced by culture medium after 6 h and cells were harvested 24–72 h following transfection for further experiments.

### Gene expression analysis

Total RNA was extracted using QIAzol (Qiagen) or the miRNeasy Mini Kit (Qiagen) following the manufacturer’s protocol. Following, 600–1000 ng RNA were reverse transcribed into cDNA via the iScript™ cDNA Synthesis Kit (BioRad) using random hexamer primers according to the manufacturer’s protocol. Relative RNA levels were quantified by quantitative real-time polymerase chain reaction (qRT-PCR) utilizing the ABsolute Blue QPCR Mix, SYBR Green, low ROX (Thermo Fisher) in a ViiA7 (ABI) or QuantStudio7 (ABI) thermocycler according to the manufacturer’s instructions with 20 ng template per reaction. Expression levels of target RNAs were normalized to housekeeping genes (*hsa_GAPDH*, *hsa_HPRT1*, *mmu_18s* and *mmu_Tbp*) and analyzed using the ΔΔC_t_ method. Primer pairs (Eurofins Genomics) utilized for qRT-PCR experiments are listed in Table 2. For circRNAs, divergent primer pairs were designed to amplify the area flanking the specific BSJ, whereas primer pairs for mRNA transcripts were designed to be intron-spanning.

**Table 2:** Primer pairs utilized for gene expression analysis.

Target	Forward (5‘→3‘)	Reverse (5ʹ→3ʹ)
*hsa_circIGF1R*	AGCCGATGTGTGAGAAGACC	GTAGCTGCGGTAGTCCTCG
*hsa_IGF1R*	GCACCATCTTCAAGGGCAATTTG	AGGAAGGACAAGGAGACCAAGG
*hsa_GAPDH*	CCAGGCGCCCAATACG	CCACATCGCTCAGACACCAT
*hsa_GUSB*	GACACCCACCACCTACATCG	CTTAAGTTGGCCCTGGGTCC
*hsa_HPRT1*	AGGACTGAACGTCTTGCTCG	GTCCCCTGTTGACTGGTCATT
*hsa_NEAT1*	GCCTTGTAGATGGAGCTTGC	TGTACCCTCCCAGCGTTTAG
*mmu_circIgf1r*	GCTGCTGGACCACAAATCG	GGTAGCTTCGGTAGTCCTCG
*mmu_18s*	GTAACCCGTTGAACCCCATT	CCATCCAATCGGTAGTAGCG
*mmu_Tbp*	TGGAATTGTACCGCAGCTTCA	CTGCAGCAAATCGCTTGGGA

For the detection of microRNAs (miRNAs), 50 ng RNA were reversed transcribed via the TaqMan MicroRNA Reverse Transcription Kit (ABI) according to the manufacturer’s instructions, utilizing the following TaqMan miRNA assays: hsa_miR-194 (#000493, ABI), hsa_miR-362 (#001273, ABI) and hsa_RNU48 (#001006, ABI). Relative miRNA levels were quantified by qRT-PCR utilizing the ABsolute Blue QPCR Mix (Thermo Fisher) in a QuantStudio7 (ABI) thermocycler according to the manufacturer’s instructions with 2 ng template per reaction. Expression levels of target *hsa_miR-194* and *hsa_miR-362* were normalized to expression levels of the housekeeping snoRNA *hsa_RNU48* and analyzed using the ΔΔC_t_ method.

### Statistics

Statistical analysis was conducted with GraphPad Prism (version 8, GraphPad). Normal distribution of data sets was examined using Anderson-Darling, D’Agostino-Pearson and Shapiro-Wilk tests. If data sets were not normally distributed according to aforementioned tests, data were logarithmized (log_10_) before application of any statistical test. Heteroscedasticity was evaluated using F test, Bartlett’s test, Brown-Forsythe test and Spearman’s test. Two-tailed Student’s t-test was used to determine statistical differences between two groups, if data sets were normally distributed and group variances were not significantly different. Statistical significance among three or more groups was determined by ordinary one-way or 2-way ANOVA if data sets were normally distributed and group variances were not significantly different. Correction for multiple comparisons was performed by applying Dunnett’s, Sidak’s, or Tukey’s post-hoc tests, depending on experimental design. All values are displayed as means ± SEM for data shown as *n* = 1, such as representative data sets, and means ± 95% confidence interval for data sets illustrated as *n* ≥ 3. Statistical significance was considered if *p* ≤ 0.05.

## Results

### Identification of circIGF1R in heart failure tissue and validation of its circular conformation

**Fig. 1:**
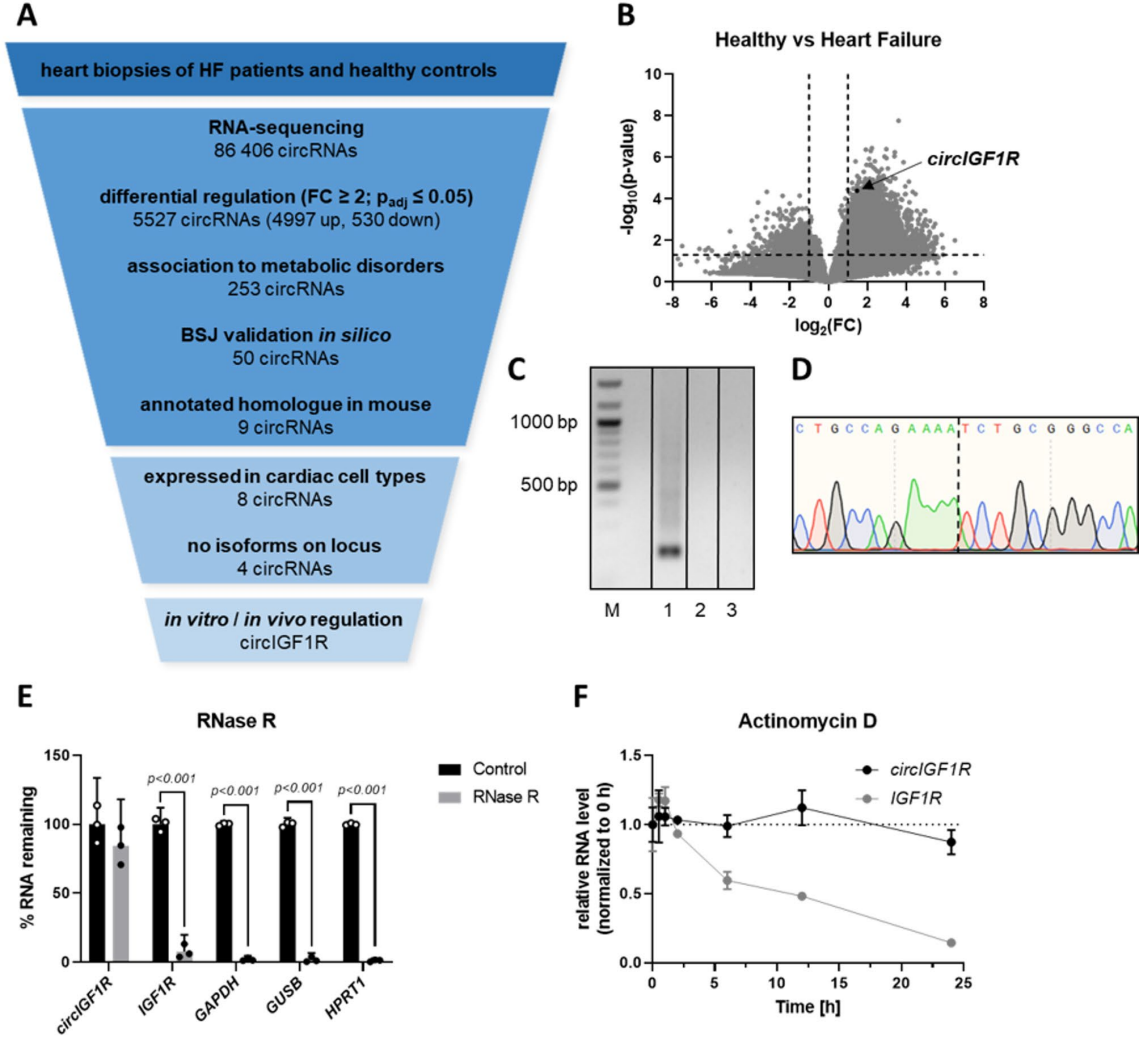
RNA sequencing identifies circIGF1R as a potential regulator of HF-progression. (**A**) Filtering strategy to identify circRNAs involved in HF progression by RNA sequencing of human HF biopsies against healthy controls (*n* = 5). (**B**) Volcano Plot of the circRNA sequencing dataset from human HF biopsies against healthy controls (*n* = 5). Cutoffs (dashed lines) were set at p_adj_ ≤ 0.05 and FC ≥ 2. (**C**) Agarose gel electrophoresis of the circIGF1R PCR product created with divergent primers. M: QuickLoad^®^ 100 bp DNA Ladder; 1: HCF cDNA; 2: HCF gDNA; 3: NTC. (**D**) Sanger sequencing of the isolated PCR product using divergent primers from HCF cDNA. Dashed line indicates the BSJ of circIGF1R. (**E**) qRT-PCR of *circIGF1R*, *IGF1R*, *GAPDH*, *GUSB* and *HPRT1* in cDNA reverse transcribed from RNase R-digested HCF RNA (*n* = 3). Data are depicted as percentage and normalized to undigested control group. Analyzed via unpaired t-test. (**F**) qRT-PCR of *circIGF1R* and *IGF1R* in HCFs after actinomycin D treatment. Data are depicted as fold change and normalized to expression levels at 0 h. Representative *n* = 1 experiment from *n* = 3 shown.

Global RNA sequencing was performed using left ventricular cardiac tissue from HF patients (in comparison to that of healthy controls) to evaluate the expression patterns of dysregulated circRNAs and identify potential therapeutic targets. In total, 86,406 circRNA transcripts were identified and filtered by (I) significantly differential expression (p_adj_ ≤ 0.05, FC ≥ 2), (II) the association of their host genes to cardiometabolic disorders, (III) the in silico validation of their BSJ utilizing the integrative genomics viewer and excluding those for which their sequencing reads did not cover their BSJ, (IV) their interspecies conservation as well as (V) their conservation in pathological in vitro and in vivo models to identify significantly dysregulated circRNAs (Fig. 1A). This stringent selection pipeline resulted in circIGF1R (hsa_circ_0005035) as our lead candidate (Fig. 1A). Compared to healthy controls, circIGF1R was significantly upregulated in HF patients (Fig. 1B, Supplemental Fig. 1A). Next, we validated the circular conformation of circIGF1R via PCR amplification utilizing divergent primers in order to amplify the region around the circRNA specific BSJ of circIGF1R. Gel electrophoresis following PCR amplification as well as Sanger sequencing of the isolated PCR product confirmed the enclosed loop structure of circIGF1R (Fig. 1C,D). After validating the circular conformation, we observed resistance of circIGF1R to exonuclease-mediated digestion as well as higher stability compared to its linear counterpart IGF1R (insulin-like growth factor 1) and other mRNA molecules, as evidenced by a prolonged half-life upon actinomycin D treatment of HCFs (Fig. 1E,F). Collectively, we identified the circRNA, circIGF1R, from RNA sequencing of HF patient samples and validated its circular structure as well as its prolonged stability compared to linear RNA molecules.

### CircIGF1R is highly conserved and downregulated in HF-associated cardiac fibroblasts

**Fig. 2:**
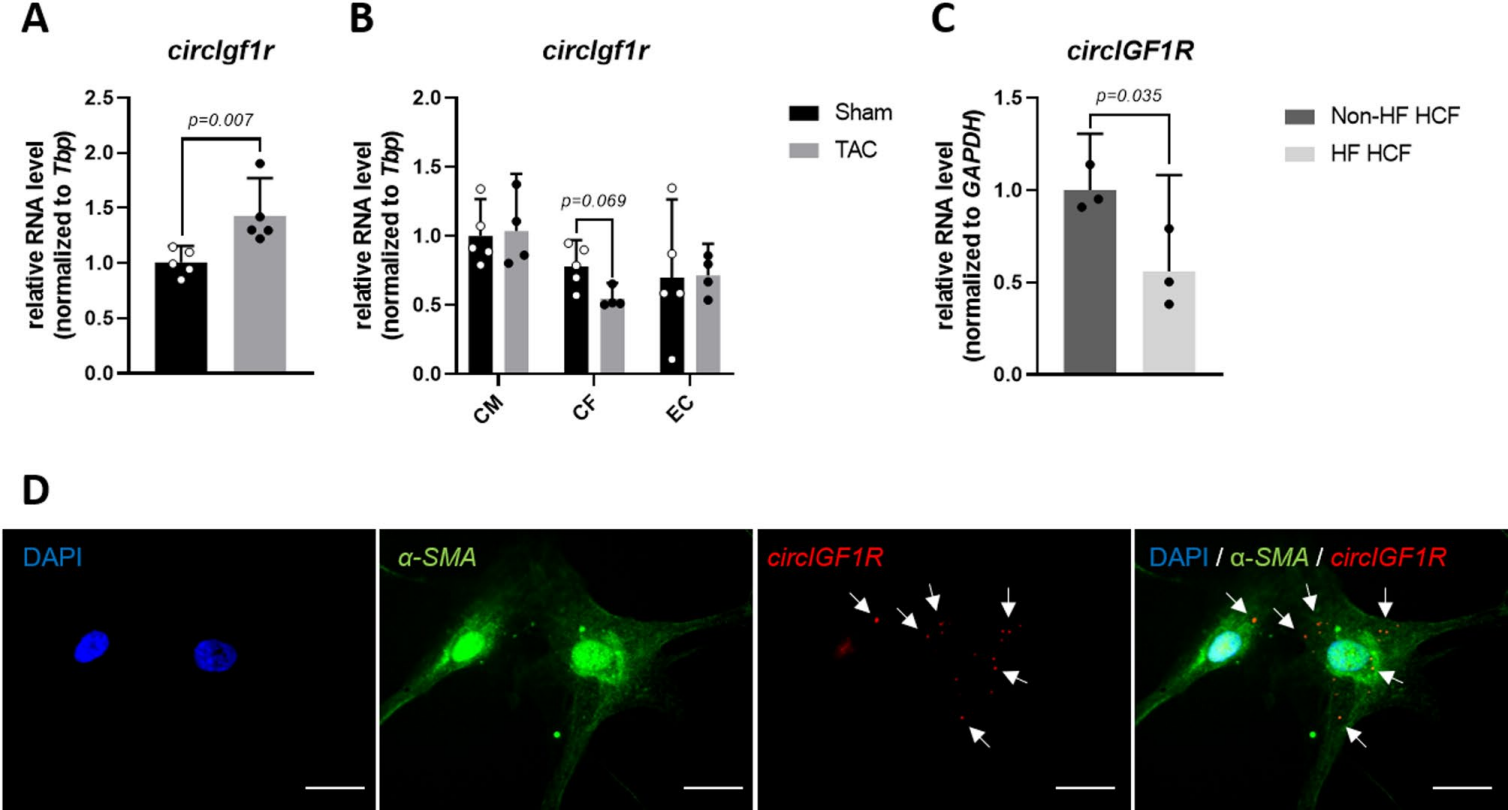
circIGF1R is dysregulated in HF-associated cardiac fibroblasts. (**A**) qRT-PCR of *circIgf1r* in adult mouse whole heart tissue after TAC surgery (*n* = 5). RNA levels of *circIgf1r* were normalized to TATA-box binding protein (*Tbp)*. Data are depicted as fold change and normalized to sham group. Analyzed via unpaired t-test. (**B**) qRT-PCR of *circIgf1r* in isolated adult mouse cardiomyocytes (CM), cardiac fibroblasts (CF) and cardiac endothelial cells (EC) after TAC surgery (*n* = 4/5). RNA levels of *circIgf1r* were normalized to *Tbp*. Data are depicted as fold change and normalized to sham group. Analyzed via matched 2-way ANOVA with Sidak’s and Tukey’s post-hoc correction. (**C**) qRT-PCR of *circIGF1R* in HF HCFs compared to non-HF HCFs (*n* = 3). RNA levels of *circIGF1R* were normalized to *GAPDH*. Data are depicted as fold change and normalized to non-HF HCF group. Analyzed via unpaired t-test. (**D**) RNA-FISH of circIGF1R in HCFs. Scale bar = 20 μm. White arrows indicate circIGF1R probe signal. α-SMA served as fibroblast cell markers and Hoechst 33342 as nuclear marker.

CircIGF1R is derived from the second exon of the protein-coding gene IGF1R and is highly conserved (> 89%) among translational relevant species, namely human, pig, mouse and rat (Supplemental Fig. 1B,C). In line with the expression in human HF, circIgf1r expression levels were elevated in a pressure-overload mouse model, which was induced via transverse aortic constriction (TAC) surgery, compared to sham animals (Fig. 2A). To evaluate its expression within the most abundant cardiac cell types (cardiomyocytes, cardiac fibroblasts and cardiac endothelial cells), we assessed the expression patterns of circIgf1r in fractionated hearts of adult mice after TAC surgery, resulting in reduced expression levels of circIgf1r in cardiac fibroblasts from TAC animals compared to sham-operated mice (Fig. 2B). Next to its considerable expression in the heart, circIgf1r was highly abundant within the brain and kidney, but with negligible expression within the aorta, liver, lung, skeletal muscle and spleen of adult mice (Supplemental Fig. 1D). Given the observed downregulation of circIgf1r in murine cardiac fibroblasts following TAC surgery, we hypothesized that circIgf1r may exert its role in the pathogenesis of HF within cardiac fibroblasts. Hence, we further investigated a potential functional role of circIGF1R in cardiac fibroblast biology in vitro. To maximize translational impact, all subsequent in vitro experiments were conducted utilizing human cardiac fibroblasts (HCFs). First, we compared the expression level of circIGF1R in non-HF-derived HCFs with those of HCFs, which were isolated from end-stage HF patients, who received implantation of a left-ventricular assist device (LVAD). Consistent with the observations in TAC mice, HCFs isolated from patients with HF (HF HCFs) exhibited significantly lower circIGF1R expression compared to HCFs derived from non-failing hearts (non-HF HCFs) (Fig. 2C). By using RNA-FISH (fluorescence in situ hybridization) with a BSJ-complementary probe (Supplemental Table 6), combined with co-staining for the fibroblast marker alpha-smooth muscle actin (α-SMA), we validated the circular conformation and cytoplasmic localization of circIGF1R (Fig. 2D). Moreover, subcellular fractionation of HCF lysates corroborated the cytoplasmic distribution of circIGF1R (Supplemental Fig. 1E). Summarizing, dysregulated expression of circIGF1R in cardiac fibroblasts might be a potential origin of HF pathogenesis.

### CircIGF1R-silenced cardiac fibroblasts exhibit increased proliferative capacity

**Fig. 3:**
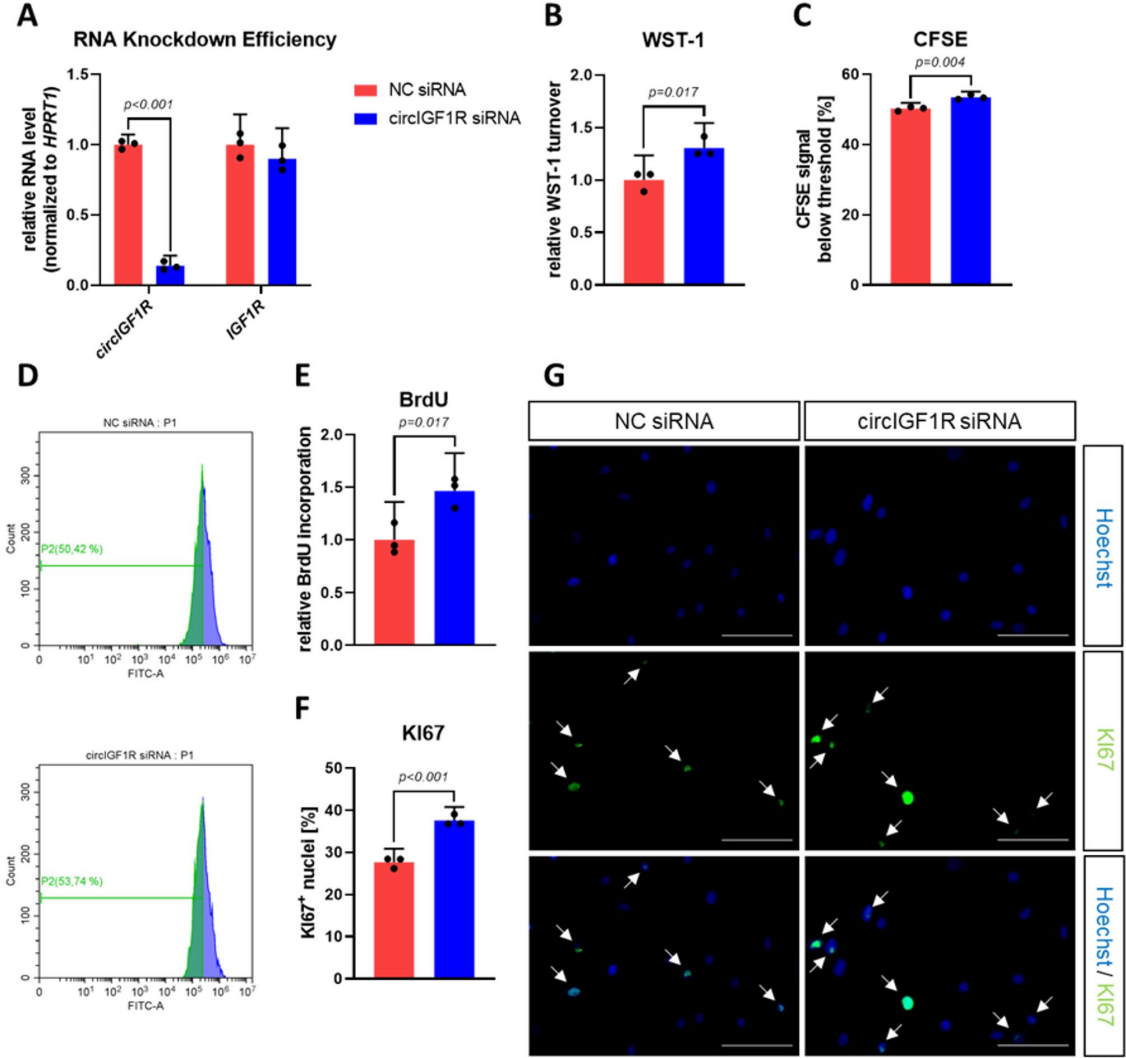
siRNA-mediated silencing of circIGF1R augments proliferation of cardiac fibroblasts. (**A**) qRT-PCR of *circIGF1R* and *IGF1R* in HCFs treated with NC siRNA or circIGF1R siRNA mix (*n* = 3). RNA levels of *circIGF1R* and *IGF1R* were normalized to *HPRT1*. Data are depicted as fold change and normalized to NC siRNA group. Analyzed via unpaired t-test. (**B**) WST-1 assay in HCFs treated with NC siRNA or circIGF1R siRNA mix (*n* = 3). Data are depicted as fold change and normalized to NC siRNA group. Analyzed via unpaired t-test. (**C**) CFSE flow cytometry in HCFs treated with NC siRNA or circIGF1R siRNA mix (*n* = 3). Analyzed via unpaired t-test. CFSE: carboxyfluorescein succinimidyl ester. (**D**) Representative histogram of CFSE flow cytometry in HCFs treated with NC siRNA or circIGF1R siRNA mix. (**E**) BrdU-ELISA in HCFs treated with NC siRNA or circIGF1R siRNA mix (*n* = 3). Data are depicted as fold change and normalized to NC siRNA group. Analyzed via unpaired t-test. (**F**) Ki67 immunofluorescence staining in HCFs treated with NC siRNA or circIGF1R siRNA mix (*n* = 3). Analyzed via unpaired t-test. (**G**) Representative images of Ki67 immunofluorescence staining in HCFs treated with NC siRNA or circIGF1R siRNA mix. White arrows indicate KI67^+^ nuclei. Scale bar = 100 μm.

To specifically evaluate the functional role of circIGF1R in the biology of cardiac fibroblasts and to exclude potential off-target effects on the mRNA of its host gene, we designed three different siRNA molecules targeting the BSJ of circIGF1R and transfected them into HCFs. A substantial knockdown of intracellular circIGF1R levels in HCFs was successfully achieved, without altering the linear counterpart IGF1R, compared to a unspecific negative control (NC) siRNA (Fig. 3A). Excessive proliferation of cardiac fibroblasts constitutes a primary mechanism underlying cardiac fibrosis and its associated adverse outcomes. Importantly, circIGF1R inhibition significantly enhanced the proliferation of HCFs, as evident by elevated turnover of WST-1 (Fig. 3B). CFSE is a fluorescent dye that dilutes after each cell division. Hence, the lower CFSE signal in HCFs following circIGF1R inhibition directly indicates increased proliferation. The pro-proliferative effect of circIGF1R silencing observed via WST-1 turnover was further confirmed by a reduction in carboxyfluorescein succinimidyl ester (CFSE) signal intensity within HCFs, as evident by an increased amount of HCFs below the set CFSE threshold (Fig. 3C,D). Similarly, enzyme-linked immunosorbent assay (ELISA)-based detection of incorporation of the base analogue 5-bromo-2’-deoxyuridine (BrdU) into the DNA with each replication cycle demonstrated increased proliferation of HCFs upon circIGF1R silencing (Fig. 3E). The nuclear protein KiI67 can be detected during each active phase of the cell cycle, while being fully absent inside the nuclei of quiescent cells. In line with our previous proliferation data, the proportion of Ki67^+^ nuclei was increased after transfection of circIGF1R siRNAs (Fig. 3F,G). Altogether, our comprehensive analysis demonstrated a promotional effect of circIGF1R silencing on cardiac fibroblast proliferation.

### Elevated glycolytic activity is associated with loss of circIGF1R and linked to cardiac fibroblast proliferation

**Fig. 4:**
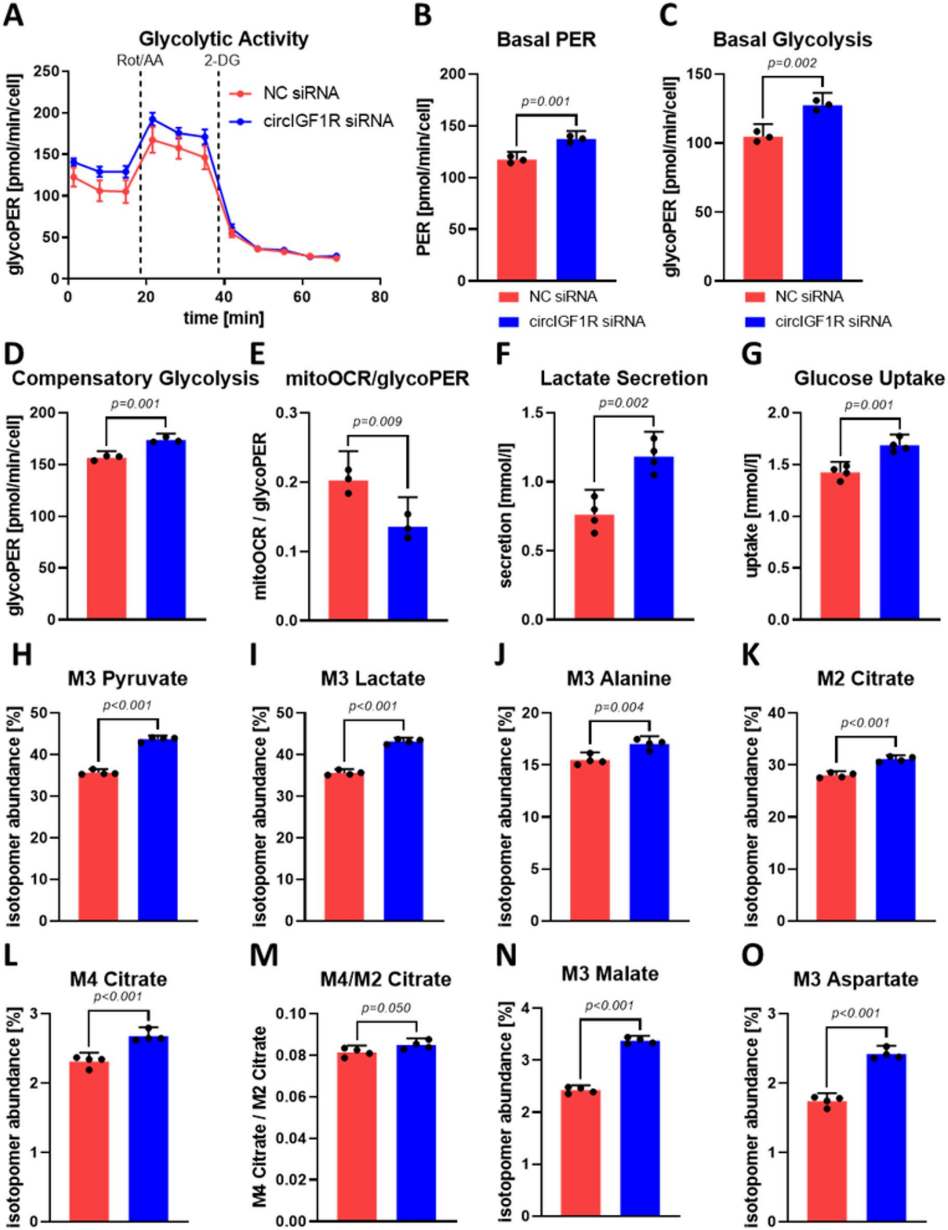
circIGF1R-silencing enhances glycolytic activity of cardiac fibroblasts. (**A**) Representative Glycolytic Rate Assay time course of HCFs treated with NC siRNA or circIGF1R siRNA mix. Dashed lines indicate injection of corresponding compounds. glycoPER: glycolytic proton efflux rate; Rot: rotenone; AA: antimycin A; 2-DG: 2-deoxyglucose. (**B**) Basal PER, (**C**) basal glycolysis, (**D**) compensatory glycolysis and (**E**) mitoOCR/glycoPER were measured via Glycolytic Rate Assay in HCFs treated with NC siRNA or circIGF1R siRNA mix (*n* = 3). Analyzed via unpaired t-test. (**F**) Lactate secretion and (**G**) glucose uptake were measured in the supernatant of HCFs treated with NC siRNA or circIGF1R siRNA mix (*n* = 4). Analyzed via unpaired t-test. (**H**) M3 pyruvate, (**I**) M3 lactate, (**J**) M3 alanine, (**K**) M2 citrate, (**L**) M4 citrate, (**M**) M4/M2 citrate, (**N**) M3 malate and (**O**) M3 aspartate were measured via GC/MS in lysates of HCFs treated with NC siRNA or circIGF1R siRNA mix (*n* = 4). Analyzed via unpaired t-test.

There is a critical role of energy metabolism in regulating cellular proliferation in various fibrotic diseases^28,29^. Notably, fibroblast activation in a diseased state necessitates a significant metabolic shift towards glycolysis. To investigate the impact of circIGF1R inhibition on this phenomenon, metabolic activity was assessed by measuring the glycolytic proton efflux rate (glycoPER), an indicator of lactate secretion resulting from glycolysis. Basal PER reflects the overall PER under resting conditions, while basal glycolysis specifically accounts for the properties of the assay buffer. Consequently, basal glycolysis isolates the PER attributed solely to glycolytic activity. Both, basal PER and basal glycolysis were significantly elevated upon circIGF1R inhibition in HCFs (Fig. 4A–C). Following the injection of complex I and complex III inhibitors rotenone and antimycin A, compensatory glycolysis describes the glycolytic activity upon abrogation of the mitochondrial electron transport chain and thereby, simulates mitochondrial dysfunction. Similar to basal PER and basal glycolysis, silencing circIGF1R resulted in increased compensatory glycolysis within HCFs (Fig. 4D). Moreover, the reduced mitoOCR (oxygen consumption rate)/glycoPER ratio describes a shift from mitochondrial respiration towards glycolysis in circIGF1R-silenced HCFs (Fig. 4E). The PER measured during the Glycolytic Rate Assay is calculated from extracellular acidification and primarily achieved by secretion of lactate. Furthermore, uptake of glucose, the primary substrate for glycolysis, is essential for maintaining glycolytic activity. Consistent with the increased glycolytic activity, analysis of the supernatant from HCFs transfected with circIGF1R siRNAs demonstrated enhanced lactate secretion and glucose import compared to control cells (Fig. 4F,G). Concurrently, we evaluated intracellular glucose trafficking via gas GC/MS (chromatography/mass spectrometry) of HCF lysates upon cultivation within medium containing ^13^C-labelled instead of ^12^C-glucose, allowing tracing of intracellular metabolization (Supplemental Fig. 1F). CircIGF1R inhibition resulted in enrichment of the glycolysis end-product M3 pyruvate as well as its following products M3 lactate and M3 alanine (Fig. 4H–J). Similarly, the M2 and M4 isotopomers of citrate, the initial intermediate of the tricarboxylic acid (TCA) cycle, were enriched in HCFs upon circIGF1R inhibition (Fig. 4K,L). The M4/M2 citrate ratio represents the efficiency of the TCA cycle and exhibited a modestly but significant elevation in circIGF1R-silenced HCFs (Fig. 4M). Consequently, other TCA cycle intermediates and byproducts, namely M3 malate and M3 aspartate, which are synthesized from pyruvate via oxaloacetate, were also increased after silencing of circIGF1R in HCFs (Fig. 4N,O). In summary, increased glucose uptake and the enriched ^13^C-labelled isotopomers of glycolysis end products implies an enhanced efficiency of glycolysis. These findings indicate enhanced cellular glucose consumption via glycolysis, resulting in elevated pyruvate and lactate production, as evident by increased glycolytic activity and lactate secretion. More interestingly, the pro-proliferative effect of silencing circIGF1R in HCFs was abolished by inhibiting glycolysis with the hexokinase inhibitor 2-deoxyglucose (2-DG). Inhibition of glycolysis exceedingly reduced BrdU incorporation and the number of Ki67^+^ HCFs, confirming the link between glycolysis and cardiac fibroblast proliferation (Supplemental Fig. 2A–C). However, circIGF1R knockdown failed to rescue the proliferation of HCFs upon glycolysis inhibition, as demonstrated by negligible restored BrdU incorporation and unaltered levels of Ki67^+^ HCFs (Supplemental Fig. 2A–C). These results suggest that circIGF1R inhibition promotes fibroblast proliferation through a mechanism dependent on glycolysis regulation. Collectively, these data highlight a potential role of circIGF1R in controlling glycolytic activity of cardiac fibroblasts, an essential prerequisite in cardiac fibroblast proliferation, which is a key characteristic of cardiac fibrosis.

### Recombinant circIGF1R mimics attenuate pathological proliferation of HF-derived cardiac fibroblasts

**Fig. 5:**
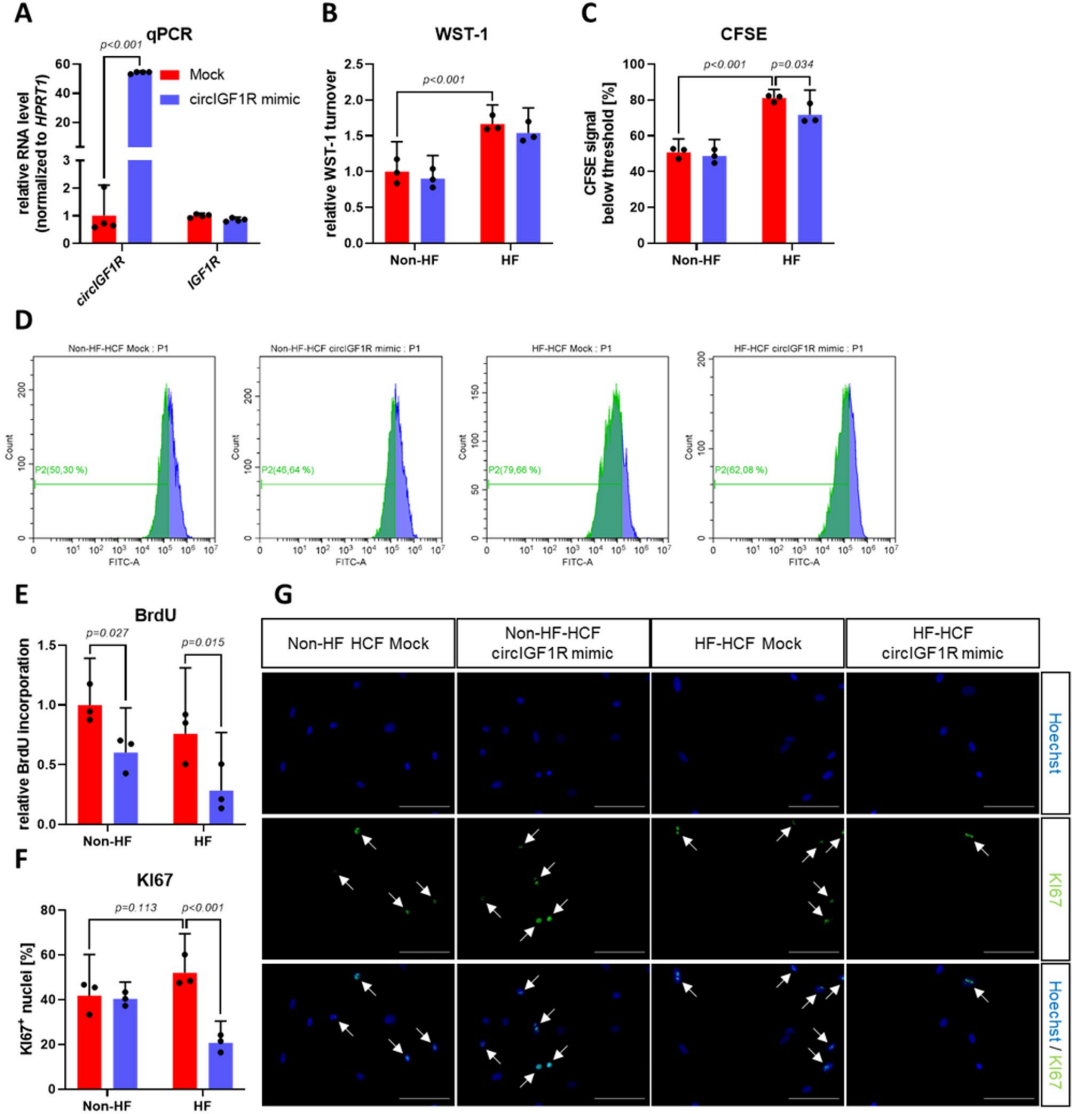
circIGF1R mimics mitigate enhanced proliferation of HF-derived cardiac fibroblasts. (**A**) qRT-PCR of *circIGF1R* and *IGF1R* in non-HF and HF HCFs treated with mock or circIGF1R mimics (*n* = 4). RNA levels of *circIGF1R* and *IGF1R* were normalized to *HPRT1*. Data are depicted as fold change and normalized to mock group. Analyzed via unpaired t-test. (**B**) WST-1 assay in non-HF and HF HCFs treated with mock or circIGF1R mimics (*n* = 3). Data are depicted as fold change and normalized to non-HF mock group. Analyzed via 2-way ANOVA with Sidak’s post-hoc correction. (**C**) CFSE flow cytometry in non-HF and HF HCFs treated with mock or circIGF1R mimics (*n* = 3). Analyzed via 2-way ANOVA with Sidak’s post-hoc correction. (**D**) Representative histogram of CFSE flow cytometry in non-HF and HF HCFs treated with mock or circIGF1R mimics. (**E**) BrdU-ELISA in non-HF and HF HCFs treated with mock or circIGF1R mimics (*n* = 3). Data are depicted as fold change and normalized to non-HF mock group. Analyzed via 2-way ANOVA with Sidak’s post-hoc correction. (**F**) Ki67 immunofluorescence staining in non-HF and HF HCFs treated with mock or circIGF1R mimics (*n* = 3). Analyzed via 2-way ANOVA with Sidak’s post-hoc correction. (**G**) Representative images of Ki67 immunofluorescence staining in non-HF and HF HCFs treated with mock or circIGF1R mimics. White arrows indicate Ki67^+^ nuclei. Scale bar = 100 μm.

In order to assess the therapeutic potential of circIGF1R, we produced recombinant circIGF1R mimics via in vitro transcription (IVT) and facilitated circularization utilizing a BSJ-complementary DNA splint. Their length was confirmed via denaturing agarose gel electrophoresis (Supplemental Fig. 3A). Moreover, PCR with divergent primers successfully amplified the region flanking the BSJ and Sanger sequencing validated the correct BSJ of our circIGF1R mimics as well as their circular conformation (Supplemental Fig. 3B,C). CircIGF1R mimics successfully overexpressed intracellular circIGF1R in HCFs without affecting mRNA levels of its linear counterpart IGF1R (Fig. 5A). As expected, proliferation of fibroblasts was significantly enhanced in HF HCFs compared to non-HF HCFs (Fig. 5B–D). While circIGF1R overexpression significantly attenuated this increased proliferation rate seen in fibroblasts derived from HF patients, as demonstrated by decreased turnover of WST-1 and increased intracellular CFSE fluorescence intensity (Fig. 5B–D). However, circIGF1R overexpression showed no effects on proliferation of non-HF HCFs, with the exception of BrdU incorporation, suggesting a more pronounced effect of circIGF1R on pathological cardiac fibroblasts (Fig. 5B–E). CircIGF1R mimics reduced BrdU incorporation of non-HF and HF HCFs to the similar extent (Fig. 5E). Consistent with WST-1 turnover and intracellular CFSE fluorescence signal, the amount of Ki67^+^ nuclei was also increased in HF HCFs compared to non-HF HCFs (Fig. 5F,G). Notably, this increase was reversed upon transfection with circIGF1R mimics (Fig. 5F,G). Summarizing, circIGF1R overexpression exhibits an anti-proliferative effect on the pathologically increased proliferation of HF-derived cardiac fibroblasts. This effect was even more pronounced within HF HCFs compared to non-HF HCFs, further strengthening the potential of circIGF1R as a therapeutic target in the therapy of cardiac fibrosis.

### Elevated glucose metabolism in HF-associated cardiac fibroblasts is rescued by circIGF1R mimics

**Fig. 6:**
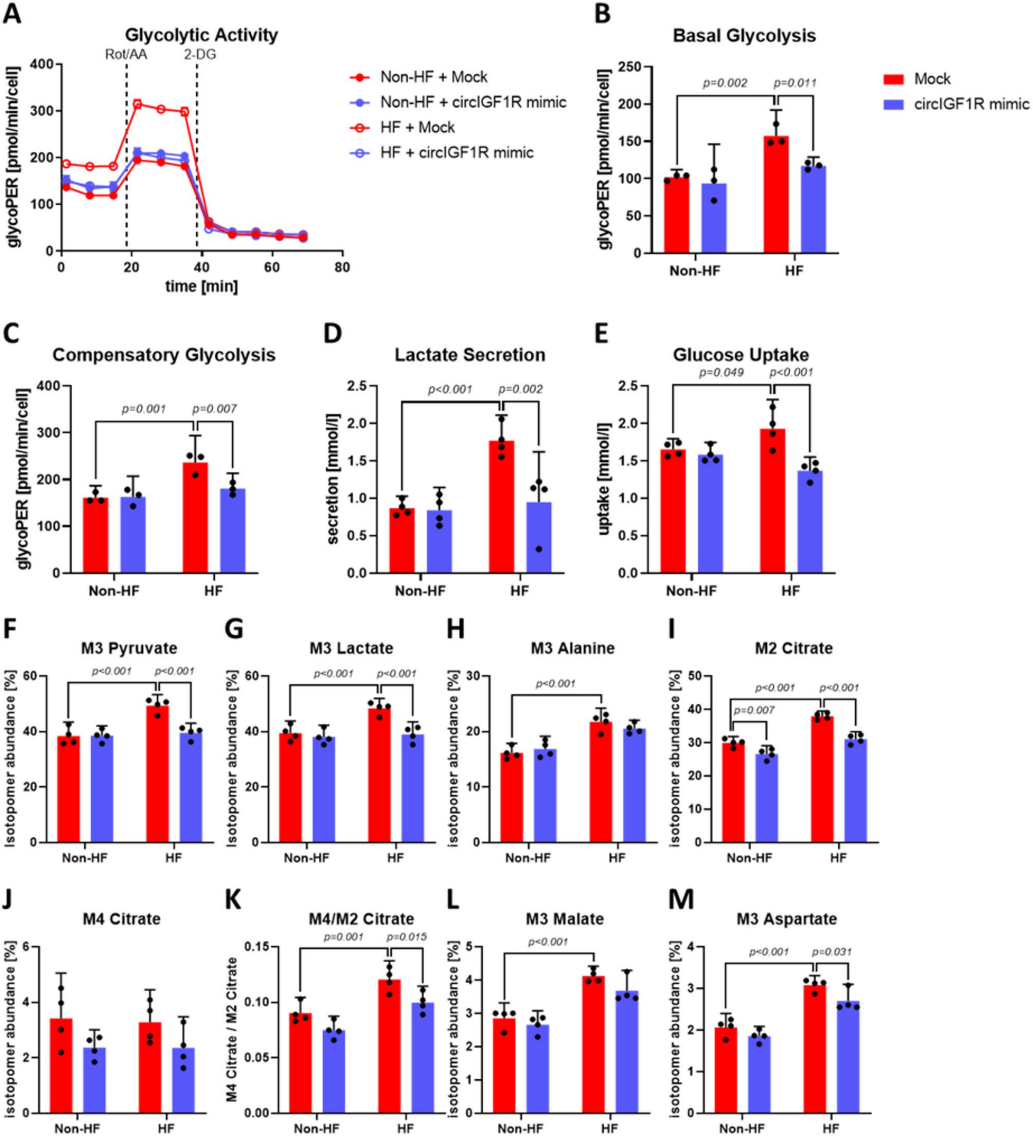
circIGF1R mimics attenuate increased glycolytic activity in HF-associated cardiac fibroblasts. (**A**) Representative Glycolytic Rate Assay time course of non-HF and HF HCFs treated with mock or circIGF1R mimics. Dashed lines indicate injection of corresponding compounds. (**B**) Basal glycolysis and (**C**) compensatory glycolysis were measured via a Glycolytic Rate Assay in non-HF and HF HCFs treated with mock or circIGF1R mimics (*n* = 3). Analyzed via 2-way ANOVA with Sidak’s post-hoc correction. (**D**) Lactate secretion and (**E**) glucose uptake were measured in supernatants of non-HF and HF HCFs treated with mock or circIGF1R mimics (*n* = 4). Analyzed via 2-way ANOVA with Sidak’s post-hoc correction. (**F**) M3 pyruvate, (**G**) M3 lactate, (**H**) M3 alanine, (**I**) M2 citrate, (**J**) M4 citrate, (**K**) M4/M2 citrate, (**L**) M3 malate and (**M**) M3 aspartate were measured via GC/MS in lysates of non-HF and HF HCFs treated with mock or circIGF1R mimics (*n* = 4). Analyzed via 2-way ANOVA with Sidak’s post-hoc correction.

During HF progression, and concurrent to exaggerated proliferation, activated cardiac fibroblasts exhibit increased glycolytic activity. Given the association between enhanced glycolysis and increased proliferation, which was demonstrated upon circIGF1R suppression, we also assessed glycolytic activity in both non-HF and HF HCFs upon transfection with circIGF1R mimics. While our circIGF1R knockdown model revealed increased glycolysis, circIGF1R mimics attenuated enhanced basal glycolysis as well as the elevated compensatory glycolysis of HF HCFs, suggesting a regulatory role of circIGF1R in metabolism of HCFs (Fig. 6A–C). In line, circIGF1R mimics attenuated both increased lactate secretion as well as enhanced import of glucose of HF HCFs (Fig. 6D,E). Tracing of intracellular glucose trafficking with a ^13^C-labelled glucose tracer demonstrated an attenuation of the enhanced utilization of glucose for glycolysis in HF HCFs following transfection of circIGF1R mimics as evidenced by decreased fractions of the isotopomers M3 pyruvate and M3 lactate (Fig. 6F,G). Comparably, M3 alanine levels were elevated in HF HCFs, but rescuing this increase with circIGF1R mimics was not significant (Fig. 6H). Moreover, increased levels of M2 citrate in HF HCFs were attenuated upon transfection with circIGF1R mimics (Fig. 6I). As M4 citrate levels remained unchanged following transfection of HF HCFs with circIGF1R mimics, the elevated M4/M2 citrate ratio in HF HCFs was diminished, indicating decreased TCA efficiency (Fig. 6J,K). Similarly, circIGF1R mimics reduced the enhanced isotopomer abundance of M3 aspartate in HF HCFs, whereas the proportion of M3 malate remained unchanged by circIGF1R mimics (Fig. 6L,M). Consistent with prior inhibition findings, the anti-proliferative activity induced by circIGF1R overexpression in HF-HCFs was fully abrogated upon glycolytic inhibition via 2-DG treatment. Glycolysis blockade profoundly attenuated BrdU incorporation and markedly decreased the proportion of Ki67^+^ HCFs, confirming the critical link between glycolysis and cardiac fibroblast proliferation (Supplemental Fig. 4A–C). Interestingly, under glycolysis suppression, circIGF1R overexpression failed to elicit additional anti-proliferative effects, as evidenced by the absence of significant further reductions in BrdU incorporation and unchanged Ki67^+^ HCF populations (Supplemental Fig. 4A–C). These findings support the potential therapeutic overexpression of circIGF1R to suppress pathological proliferation of cardiac fibroblasts through metabolic modulation.

Collectively, our combined observations of glycolytic rate measurement and intracellular glucose tracing suggest a protective role of circIGF1R by alleviating the enhanced glycolytic activity of HF-associated cardiac fibroblasts through the combined regulation of glucose import and through supporting removal of glycolysis intermediates for other metabolic pathways. In conclusion, these data suggest an anti-proliferative and anti-fibrotic role of circIGF1R by reducing glycolytic activity and suppressing glucose uptake in HF-associated activation of cardiac fibroblasts.

### CircIGF1R reduces glycolytic activity via AZGP1

**Fig. 7:**
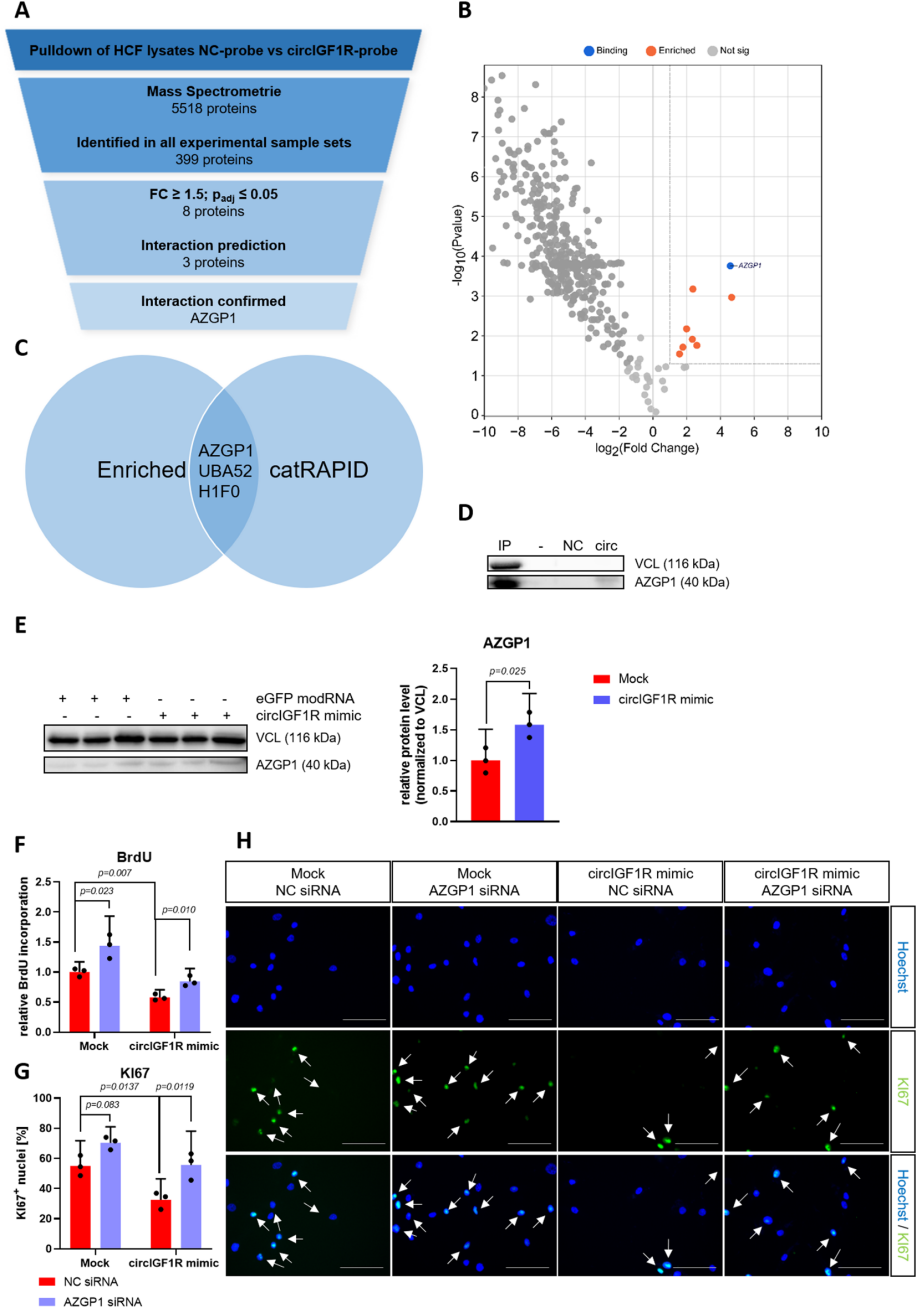
RNA pulldown identifies multiple protein interaction partners of circIGF1R. (**A**) Filtering strategy to identify protein interaction partners of circIGF1R in non-HF-derived HCFs upon RNA pulldown with circIGF1R-specific probe NC probe (*n* = 4). (**B**) Volcano Plot of RNA pulldown experiments from lysates of non-HF-derived HCFs treated with circIGF1R-specific probe or NC probe (*n* = 4). Dashed lines indicate thresholds (FC ≥ 1.5; *p*_*adj*_ ≤ 0.05). (**C**) Venn diagram showing the overlapping proteins between in silico interaction prediction via catRAPID and significantly-enriched proteins. (**D**) Western Blot of non-HF-derived HCF lysates after RNA-pulldown with circIGF1R-specific probe or NC probe (*n* = 1). IP: Input; NC: NC probe; circ: circIGF1R probe. (**E**) Western Blot of HF-derived HCFs treated with mock or circIGF1R mimics including quantified protein levels (*n* = 3). VCL: Vinculin. (**F**) BrdU-ELISA in HF-HCFs transfected with either NC siRNA or AZGP1 siRNA, followed by treatment with mock or circIGF1R mimics (*n* = 3). Data are depicted as fold change and normalized to the cotreatment group receiving mock and NC siRNA. Analyzed via 2-way ANOVA with Sidak’s post-hoc correction. (**G**) Ki67 immunofluorescence staining in HF HCFs transfected with either NC siRNA or AZGP1 siRNA, followed by treatment with mock or circIGF1R mimics (*n* = 3). Analyzed via 2-way ANOVA with Sidak’s post-hoc correction. (**H**) Representative images of Ki67 immunofluorescence staining in HF HCFs transfected with either NC siRNA or AZGP1 siRNA, followed by treatment with mock or circIGF1R mimics. White arrows indicate Ki67^+^ nuclei. Scale bar = 100 μm.

To unveil the specific mechanism by which circIGF1R impairs glycolytic activity and proliferation of cardiac fibroblasts, we designed BSJ-complementary DNA probes to pulldown circIGF1R together with its specific interaction partners (Supplemental Table 7). Following, potential protein binding partners of circIGF1R were identified via MS. Out of 5518 identified proteins, 399 were detected in all experimental sample sets (Fig. 7A and Supplemental Table 8). Proteins were further selected based on their enrichment in the circIGF1R probe group compared to HCFs lysates as well as the NC probe group (FC ≥ 1.5; *p*_*adj*_ ≤ 0.05), resulting in eight significantly enriched proteins in the circIGF1R probe group (Fig. 7A,B). Next, in silico prediction of circIGF1R against the RNA binding proteome via catRAPID resulted in three significantly enriched proteins: AZGP1 (alpha-2-glycoprotein 1, zinc-binding), UBA52 and H1F0 (Fig. 7A,C). Out of these candidates, solely AZGP1 is filtered by both identification via MS as well as relevant functions linked to glucose uptake and glycolysis^30,31^. Moreover, several independent investigations have already demonstrated an anti-proliferative function of AZGP1^32–34^. Interaction of circIGF1R and AZGP1 was validated via Western Blot of HCF lysates following RNA pulldown with our circIGF1R-specific probe (Fig. 7D). Consequently, AZGP1 was enriched in HCFs treated with circIGF1R mimics compared to mock-treated cells (Fig. 7E). To further study the interaction between AZGP1 and circIGF1R on fibroblast proliferation, we have employed siRNA-mediated AZGP1 inhibition, followed by treatment with mock or circIGF1R overexpression in HF-HCFs. Consistent with previous findings, circIGF1R overexpression demonstrated significant reductions in BrdU incorporation (Fig. 7F) and Ki67^+^ populations of HF HCFs (Fig. 7G,H). Importantly, these anti-proliferative effects were reversed upon suppression of AZGP1 protein expression (Fig. 7F–H). These results indicate that AZGP1 inhibition disrupts the anti-proliferative function of circIGF1R, underscoring the critical role of AZGP1-circIGF1R interaction in regulating cardiac fibroblast proliferation. Further investigation is required to elucidate the precise molecular mechanisms underlying this interaction and its contribution to pathological pathways during fibroblast proliferation and fibrosis progression. In summary, RNA pulldown resulted in identification of AZGP1 as a potential interaction partner of circIGF1R, contingently representing the missing link between circIGF1R and its inhibitory effect on glycolysis as well as its anti-proliferative function in cardiac fibroblasts.

## Discussion

Despite the high socioeconomic burden of cardiac fibrosis, a key feature of HF, current treatment options remain limited. Recent studies suggest that circRNAs hold therapeutic promise for several cardiovascular disease, including doxorubicin-induced cardiotoxicity^18 ^cardiac fibrosis^20,21^ and hypertrophic cardiomyopathy^35^. However, therapeutic intervention targeting cardiac fibroblasts, the primary effector cell type driving cardiac fibrosis, are still underdeveloped. In this study, we demonstrate an anti-proliferative function of circIGF1R in cardiac fibroblasts, establishing it as a novel therapeutic target for cardiac fibrosis. Primarily, circIGF1R attenuates excessive pathological proliferation of HF-associated cardiac fibroblasts by inhibiting glucose uptake into the cell and thereby reducing glycolytic activity.

Aiming to find a new therapeutic approach for HF, we performed circRNA sequencing of end-stage HF patients, resulting in circIGF1R emerging from our stringent bioinformatics approach and experimental criteria as the most promising candidate. CircIGF1R is derived from the well-characterized protein-coding gene IGF1R. While IGF1R is already known to be involved in energy metabolism^36 ^circRNAs have been reported to exhibit host gene-independent expression patterns and functions^18,35,37^. In this study, we have utilized BSJ-complementary siRNAs as well as IVT to specifically target circIGF1R without affecting the linear transcript of IGF1R, thereby allowing the assessment of circIGF1R independently from the expression of its host gene. CircIGF1R demonstrates a broad spectrum of species conservation across small (mouse, rat) and large (porcine) animal models, as well as human, highlighting its potential for robust pre-clinical development. While circIGF1R exhibits widespread expression across various cell types within cardiac tissue, its dysregulation following HF appears to be fibroblast-specific. In a murine model of cardiac fibrosis induced by TAC, circIGF1R expression remained unchanged in cardiomyocytes and endothelial cells, but exhibited reduced levels in fibroblasts, suggesting a fibroblast-specific role for circIGF1R in the context of HF. Notably, circIGF1R exhibited dysregulated expression levels in HF-associated HCFs compared to healthy control HCFs. The contradictory regulation patterns of circIGF1R in whole heart tissue of TAC-operated mice compared to HF-associated fibroblasts might originate in other cardiac cell types exhibiting highly elevated levels of circIGF1R following pressure-overload-induced HF. However, identification of these cell types remained elusive due to technical limitations of our isolation protocol, wherefore we solely focused on the main cardiac cell types in this study.

To elucidate the role of circIGF1R in fibroblast activation and proliferation, we employed siRNA-mediated silencing to achieve loss-of-function, followed by comprehensive functional assays – including substrate turnover, flow cytometry, ELISA as well as immunofluorescence staining. Fibrotic tissues exhibit a tight coupling between cellular metabolism and fibroblast activation. More precisely, activated fibroblasts undergo metabolic reprogramming, prioritizing rapid proliferation and scar formation^13,38^. Particularly, glycolysis is vital for proliferation, not only through generation of energy equivalents like ATP, but also by providing essential building blocks in terms of glycolysis intermediates to fuel synthesis of fatty acids, nonessential amino acids, and nucleotides^39^. Chen et al.. recently demonstrated that suppressing glycolysis of cardiac fibroblasts attenuated proliferation and reversed the activated phenotype upon transforming growth factor beta (TGF-β) stimulation^13^. This finding suggests a potential link between glycolytic activity and the observed pro-proliferative effects of silencing circIGF1R. Building upon this, we further examined glycolytic activity, glucose uptake and intracellular glucose trafficking. Our findings revealed that loss of circIGF1R significantly enhanced glycolytic activity by increasing glucose import and altering intracellular glucose trafficking. Hence, the increased synthesis of pyruvate can be utilized to generate amino acids such as alanine and aspartate, which are needed for protein biosynthesis and cellular proliferation^11^. More interestingly, circIGF1R siRNAs did not rescue the decreased fibroblast proliferation following abrogation of glycolysis by the hexokinase inhibitor 2-DG, indicating an essential role of glycolysis in mediating the pro-proliferative function of circIGF1R siRNAs.

Following our discovery of the pro-proliferative role of circIGF1R inhibition, we hypothesized that circIGF1R overexpression might conversely suppress abnormal fibroblast proliferation in disease states. To investigate this gain-of-function, we employed synthetic recombinant circIGF1R mimics generated via IVT for overexpression in patient-derived fibroblasts from individuals with HF, characterized by reduced endogenous circIGF1R levels. In contrast to circIGF1R inhibition, IVT-mediated circIGF1R overexpression effectively rescued the elevated proliferation, glycolytic activity, glucose import and altered glucose trafficking observed in HF HCFs. Notably, the effect was significantly more pronounced in HF HCFs compared to non-HF HCFs, further highlighting the therapeutic potential of circIGF1R overexpression in the context of HF-associated fibrosis.

Circular RNAs often exert their functions by interacting with RNA-binding proteins or microRNAs. To delve deeper into the anti-fibrotic mechanisms of circIGF1R, we conducted RNA pulldown assays coupled with mass spectrometry. Combined with in silico predictions, we identified the protein, AZGP1, as the most promising binding partner of circIGF1R. Previous studies have shown that AZGP1 exerts anti-fibrotic and cardioprotective effects by preventing fibroblast activation via regulation of TGF-β signaling^40,41^. Notably, our findings revealed that circIGF1R mimics significantly upregulate the protein level of AZGP1, suggesting that the anti-fibrotic effects of circIGF1R mimics may be further enhanced by their interaction with AZGP1. Next to AZGP1, S100A8 (S100 Calcium Binding Protein A8) and EMILIN1 were significantly enriched by our circIGF1R-specific probe in HCF lysates. S100A8 is known to reduces glucose metabolism and inhibits proliferation^42,43^, while EMILIN1 inhibition is associated with hyper-proliferation of fibroblasts^44,45^. Furthermore, H1F0, SERPINB3, SERPINB12 and UBA52 were also significantly enriched; these proteins are already recognized for their roles in regulating proliferation^46–49^.

A competing endogenous role of circRNAs for miRNAs has first been reported by Hansen et al.. in 2013, by demonstrating over 70 conserved binding sites for miR-7 within the circRNA ciRS-7^50^. Despite many studies demonstrating miRNA sponging by various circRNAs, most circRNAs only possess a few binding sites for their target miRNA, unlike the extensive binding sites found for miR-7 within ciRS-7^50^. CircIGF1R has previously been implicated in the pathophysiology of colorectal cancer and psoriasis, where it regulates proliferation of cancer cells and keratinocytes by sponging of miR-362-5p^51^ and miR-194-5p^52^ respectively. Moreover, circIgf1r has been reported to promote the proliferation of primary mouse cardiomyocytes by sponging miR-362-5p and thereby suppressing its function to induce degradation of pro-proliferative *Phf3* transcripts^53^. Consequently, a non-targeted therapeutic application of circIGF1R could induce beneficial effects on HF pathophysiology by exerting a dual effect: counteracting the detrimental loss of cardiomyocytes as well as attenuating cardiac fibroblast proliferation to mitigate cardiac fibrosis and myocardial stiffening. However, sequence analysis of circIGF1R resulted in only one predicted binding site for miR-194, and no predicted binding sites for miR-362^54^. Additionally, qRT-PCR analysis did not detect significant upregulation of miR-194 or miR-362 upon circIGF1R knockdown in cardiac fibroblasts (Supplemental Fig. 5). Hence, the inhibitory role of circIGF1R on glucose import as well as its anti-proliferative function in cardiac fibroblasts is highly likely to be mediated by an alternative interaction partner, rather than through direct interaction with miRNAs.

Although results obtained in this study revealed a potentially novel regulatory role of circIGF1R in anti-fibroblast proliferation and glycolysis modulation in the context of heart failure, our current findings are limited to in vitro systems. Therefore, more investigation is needed to further evaluate its therapeutic potential. To substantiate the role of circIGF1R, several key steps are warranted. First, the link between the anti-proliferative effects of circIGF1R and key processes in fibrosis development, such as fibroblast migration and extracellular matrix (ECM) production, should be elucidated. In line with that, in vivo validation using animal models of cardiac fibrosis or pressure-overload-induced heart failure is critical to establish its functional relevance. Recent advances in RNA therapeutics, including FDA-approved IVT mRNA vaccines^55 ^have demonstrated the feasibility of overcoming immune stimulatory effects through base-modified nucleosides^56^ and achieving tissue-specific delivery via lipid nanoparticles (LNPs). Fibroblast-specific LNPs have shown therapeutic promise in preclinical models of organ fibrosis^57,58^ and could be explored for circIGF1R delivery. Second, elucidating the molecular mechanism underlying circIGF1R function, particularly its interaction with the RNA-binding protein AZGP1, may uncover downstream effectors responsible for glycolytic control and cell proliferation, potentially identifying additional druggable targets. Finally, translational studies using patient-derived fibrotic cardiac tissues would provide critical insights into its clinical relevance. Living myocardial slices (LMS), which retain the native multicellular architecture and can be cultured under controlled conditions for months^59 ^represent a valuable platform for investigating cardiac pathophysiology and therapeutic targets. Specifically, LMS derived from patients with heart failure^60^ provide an opportunity to study the translational relevance of circIGF1R modulation in fibrosis associated with heart failure. Together, these future directions will be essential in elucidating the role of circIGF1R in fibroblast activation and the progression of fibrosis in heart failure, as well as in evaluating its potential as therapeutic intervention.

Overall, this study presents a comprehensive analysis of glucose metabolism and proliferation in patient-derived cardiac fibroblasts. We demonstrate the potential of circIGF1R (hsa_circ_0005035) as a novel therapeutic approach for cardiac fibrosis and HF. CircIGF1R acts by alleviating the pathologically elevated carbohydrate metabolism through regulation of glucose uptake by interaction with AZGP1 and consequently, attenuates excessive proliferation of cardiac fibroblasts to alleviate cardiac fibrosis. To our knowledge, this is the first study to establish the role of circRNAs in cardiac energy metabolism and its connection to fibroblast proliferation. Furthermore, the expression of circIGF1R in other organs susceptible to tissue fibrosis, namely the kidney, highlights its potential as a promising target for broader anti-fibrotic therapies.

## Conclusion

CircIGF1R is ubiquitously expressed throughout all main cardiac cell types but solely downregulated within HF-associated cardiac fibroblasts. Silencing circIGF1R in cardiac fibroblasts resulted in exaggerated proliferation and enhanced glycolytic activity originating from an increased glucose uptake and altered intracellular glucose trafficking. Consequently, circIGF1R overexpression diminished these pathological effects within HF-associated HCFs by attenuating glucose import into cardiac fibroblasts, thereby attributing a therapeutic potential of circIGF1R in HF therapy by alleviating cardiac fibrosis via AZGP1.

## Electronic supplementary material

Below is the link to the electronic supplementary material.

Supplementary Material 1

Supplementary Material 2

Supplementary Material 3

## Data Availability

The circRNA profile generated from human healthy and heart failure samples are available in Gene Expression Omnibus (GEO) under the accession numbers GSE295478. All data generated or analyzed during this study, including the mass spectrometric data produced by CircIGF1R pull down, are available in the article and in its online supplementary material.
