# Supplementary material for: Circular RNA circIGF1R controls cardiac fibroblast proliferation through regulation of carbohydrate metabolism
Source: Sci Rep. 2025 Jun 27;15:20331. doi: 10.1038/s41598-025-07167-3 (PMC12205068; doi:10.1038/s41598-025-07167-3)
Supplement: Supplementary file 1 — Supplementary Material 1 [file 41598_2025_7167_MOESM1_ESM.pdf]

Circular RNA circIGF1R controls cardiac fibroblast proliferation through regulation of carbohydrate metabolism

Arne Schmidt[1,2.3], Kevin Schmidt[1,2.3], Sonja Groß[1], Dongchao Lu[1], Ke Xiao[1.2.3], Dimyana Neufeldt[1], Sarah Cushman[1], Nele Lehmann[1], Sabrina Thum[1], Angelika Pfanne[1], Annette Just[1], Andreas Pich[4,5], Alexander Heinz[6], Karsten Hiller[6], Wilson Lek Wen Tan[7], Roger Foo[7], Christian Bär[1,2,3,8], Mira Jung[1,*], Thomas Thum[1,6,*]

[1] Institute of Molecular and Translational Therapeutic Strategies, Hannover Medical School, Hannover, Germany

[2] Fraunhofer Institute for Toxicology and Experimental Medicine (ITEM), Hannover, Germany

[3] Fraunhofer Cluster of Excellence Immune-Mediated Diseases (CIMD), Hannover, Germany

[4] Institute of Toxicology, Hannover Medical School, Hannover, Germany

[5] Core Facility Proteomics, Institute of Toxicology, Hannover, Germany

[6] Department of Bioinformatics and Biochemistry, Braunschweig Integrated Centre of Systems Biology (BRICS), Technische Universität Braunschweig, Braunschweig, Germany

[7] Institute of Molecular and Cell Biology, A*STAR, Singapore, Singapore

[8] Center for Translational Regenerative Medicine, Hannover Medical School, Hannover, Germany

[#] Co-corresponding authors

Thomas Thum, MD, PhD
Hannover Medical School, Institute of Molecular and Translational Therapeutic Strategies
Tel: 0049-511-532-5272
Email: thum.thomas@mh-hannover.de

Mira Jung, PhD
Hannover Medical School, Institute of Molecular and Translational Therapeutic Strategies
Tel: 0049-511-532-5276
Email: jung.mira@mh-hannover.de

Supplementary figures

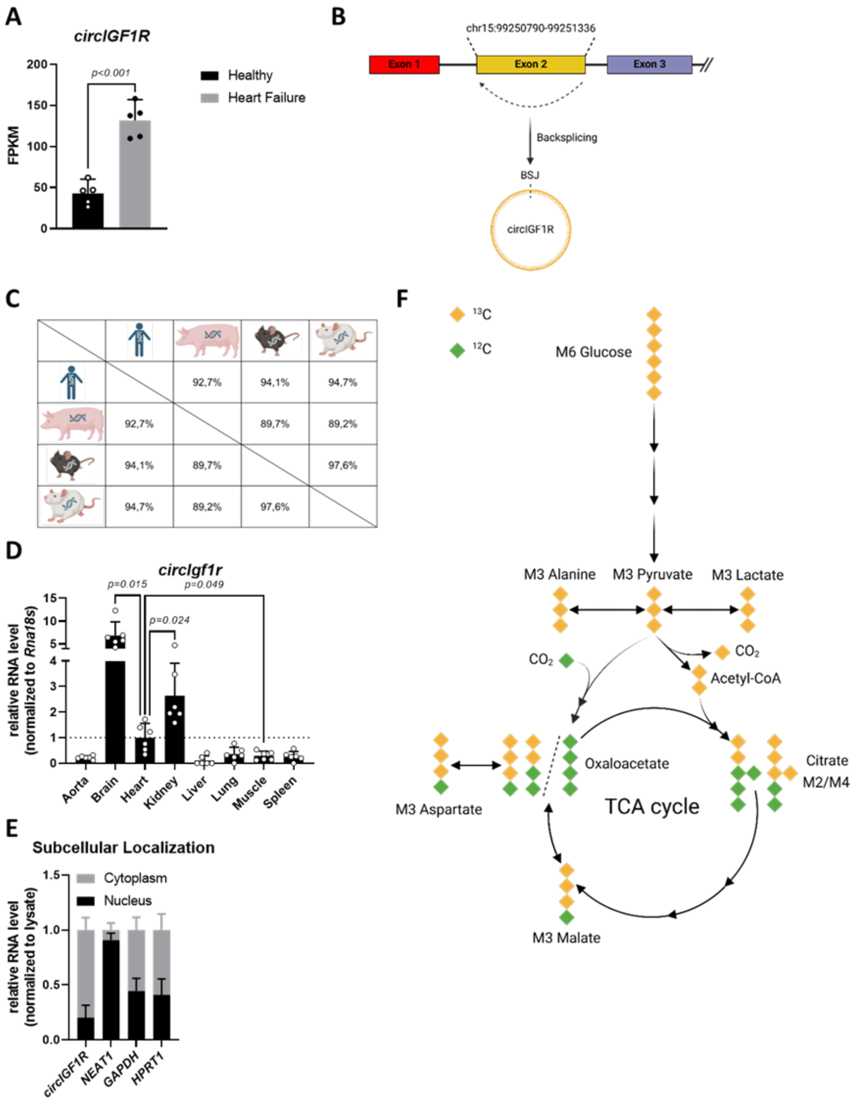

Supplementary Fig. 1: circIGF1R is regulated in human HF and highly conserved. **A** FPKM values of *circIGF1R* in human HF biopsies and healthy controls ($n = 5$). Analyzed via unpaired t-test. FPKM: fragments per kilobase million. **B** Schematic representation of the circIGF1R-encoding genomic locus within IGF1R. BSJ: backsplice junction. Created with BioRender.com. **C** Conservation matrix reporting the percentage of conservation of circIGF1R in human, pig, mouse and rat. Multiple sequence alignment was conducted using ClustalW (version 1) from the European Bioinformatics Institute[1,2]. Created with BioRender.com. **D** qRT-PCR of *circIgf1r* in aorta, brain, heart, kidney, liver, lung, skeletal muscle and spleen of adult mice ($n = 6$). RNA levels of *circIgf1r* were normalized to *Rna18s*. Data are depicted as fold change and normalized to heart group. Analyzed via matched one-way ANOVA with Dunnett's post-hoc correction. **E** qRT-PCR of *circIGF1R, NEAT1, GAPDH* and *HPRT1* in subcellular fractionated

HCF lysates ($n = 3$). RNA levels were normalized to lysate and depicted as total fractions. **F** Schematic representation of intracellular glucose trafficking. Substrates and byproducts of glycolysis and the TCA cycle were labelled utilizing a ^{13}C-labelled glucose tracer and analyzed via GC/MS. “M” indicates the number of ^{13}C-labelled atoms (yellow) within each molecule. TCA: tricarboxylic acid. Created with biorender.com.

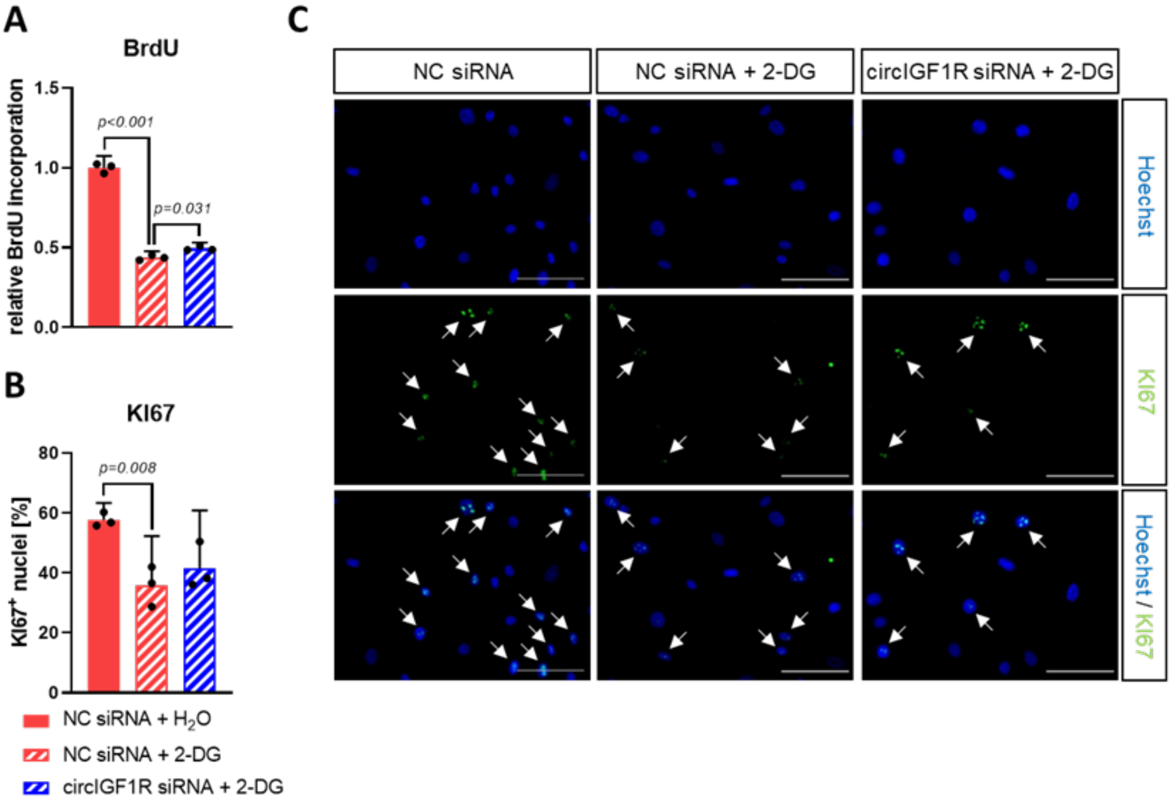

Supplementary Fig. 2: circIGF1R-silencing does not rescue the impaired proliferation of HCFs upon glycolysis inhibition. **A** BrdU-ELISA in non-HCFs treated with NC siRNA or circIGF1R siRNA mix and H_2O or 2-DG ($n = 3$). Data are depicted as fold change and normalized to NC siRNA + H_2O group. Analyzed via one-way ANOVA with Dunnett’s post-hoc correction. **B** KI67 immunofluorescence staining in non-HCFs treated with NC siRNA or circIGF1R siRNA mix and H_2O or 2-DG ($n = 3$). Analyzed via one-way ANOVA with Dunnett’s post-hoc correction. **C** Representative images of KI67 immunofluorescence staining in non-HCFs treated with NC siRNA or circIGF1R siRNA mix and H_2O or 2-DG. White arrows indicate KI67^{+} nuclei. Scale bar = 100 µm. **D** BrdU-ELISA in HF-HCFs treated with Mock or circIGF1R mimics and H_2O or 2-DG ($n = 3$). Data are depicted as fold change and

normalized to Mock + H_2O group. Analyzed via one-way ANOVA with Dunnett's post-hoc correction. **E** KI67 immunofluorescence staining in HF-HCFs treated with Mock or circIGF1R mimics and H_2O or 2-DG ($n = 3$). Analyzed via one-way ANOVA with Dunnett's post-hoc correction. **F** Representative images of KI67 immunofluorescence staining in HF-HCFs treated with Mock or circIGF1R mimics and H_2O or 2-DG. White arrows indicate $KI67^+$ nuclei. Scale bar = 100 µm.

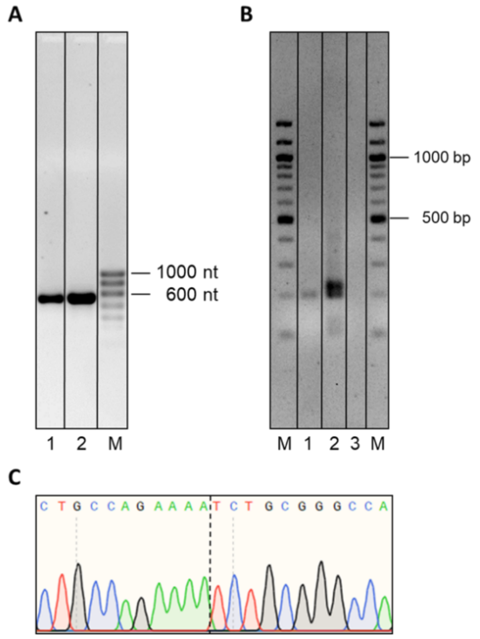

Supplementary Fig. 3: Recombinant circIGF1R mimics exhibit circular conformation. **A** Denaturing agarose gel of circularized and uncircularized circIGF1R mimics. 1: circularized circIGF1R mimics; 2: uncircularized circIGF1R mimics; M: RiboRuler RNA Ladder, Low Range. **B** Agarose gel of the PCR product created with divergent primers. M: QuickLoad® 100 bp DNA Ladder; 1: HCF cDNA; 2: circIGF1R mimics; 3: NTC. **C** Sanger sequencing of the PCR product created with divergent primers from circIGF1R mimics. Dashed line indicates the BSJ of circIGF1R mimics.

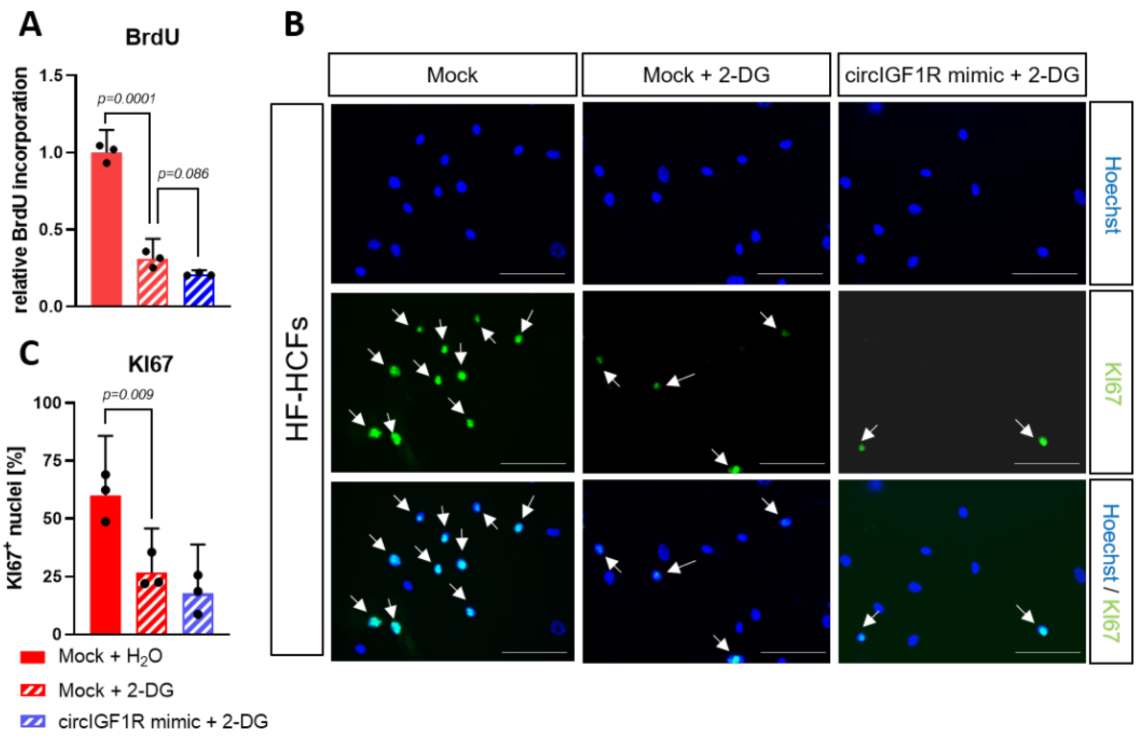

Supplementary Fig. 4: Glycolysis inhibition disturbs anti-fibrotic effects of circIGF1R overexpression. **A** BrdU-ELISA in HF-HCFs treated with Mock or circIGF1R mimics and H_2O or 2-DG (*n* = 3). Data are depicted as fold change and normalized to Mock + H_2O group. Analyzed via one-way ANOVA with Dunnett's post-hoc correction. **B** KI67 immunofluorescence staining in HF-HCFs treated with Mock or circIGF1R mimics and H_2O or 2-DG (*n* = 3). Analyzed via one-way ANOVA with Dunnett's post-hoc correction. **C** Representative images of KI67 immunofluorescence staining in HF-HCFs treated with Mock or circIGF1R mimics and H_2O or 2-DG. White arrows indicate $KI67^+$ nuclei. Scale bar = 100 µm.

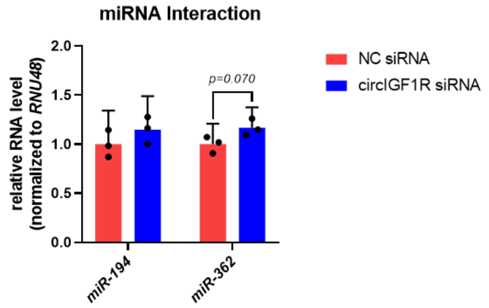

Supplementary Fig. 5: Predicted miRNA target levels remain unchanged upon circIGF1R-silencing. Taq-Man qRT-PCR of *miR-194* and *miR-362* in HCFs treated with NC siRNA or circIGF1R siRNA mix

($n = 3$). RNA levels of *miR-194* and *miR-362* were normalized to *RNU48*. Data are depicted as fold change and normalized to NC siRNA group. Analyzed via unpaired t-test.

Supplementary methods

Human heart tissue sampling

Supplementary Table 1: Clinical characteristics of HF patients

**age [years]**	**sex**	**underlying disease**	**comorbidities**	**last medication (daily dose)**
47	female	DCM	none	xipamide (1x 10 mg), carvedilol (2x 6.25 mg), captopril (3x 12.5 mg), isosorbide dinitrate (2x 40 mg), furosemide intravenously for > 3 months
56	male	DCM	NIDDM	phenprocoumon (1x 3 mg), furosemide (2x 40 mg), enalapril (1x 10 mg), glyceryl trinitrate (as required), molsidomine (1x 8 mg), ranitidine (1x 150 mg) for > 12 months
18	male	transposition of arterial trunk	none	captopril (3x 25 mg), digitoxin (2x 0.2 mg) for > 3 months
13	female	DCM	none	glyceryl trinitrate, digoxin (2x 0.075 mg), captopril (2x 12.5 mg) for > 3 months
39	male	DCM	none	digoxin (2x 0.075 mg), captopril (2x 25 mg), carvedilol

				(1x 6.25 mg), torasemide (1x 2.5 mg), spironolactone (1x 75 mg) for > 12 months

Cardiac tissue samples were collected from patients with HF[3] or healthy controls[4]. Ethical approval for the use of patient samples in this research was granted by the institutional ethics committee of the Hannover Medical School, Germany. The study was conducted according to the guidelines from the declaration of Helsinki and its amendments or comparable ethical standards. Data collection of the patient samples including written informed consent and analysis methods adhered to those established in previous studies[5]. Further information comprising age, sex, underlying diseases, comorbidities as well as the last medication of HF patients[3] are listed in Supplementary Table 1.

In vitro circRNA validation

Circularity of potential candidates was evaluated by PCR amplification using the Hot StarTaq Master Mix Kit (Qiagen) with divergent primer pairs flanking the BSJ (Table 2) according to the manufacturer's protocol using 10 ng of DNA template per reaction, followed by 1.5 % agarose (Carl Roth) gel electrophoresis (supplemented with Midori Green Advance (Biozym)) of the resulting amplicons. Therefore, the PCR product was mixed with 6x loading dye (10 mM Tris-HCl (pH 7.5, Carl Roth), 60 mM EDTA (Sigma), 60 % glycerol (Carl Roth), 0.3 % bromphenol blue (Sigma)) and loaded onto the gel together with the Quick-Load® 100 bp DNA Ladder (New England Biolabs). Subsequently, DNA fragments of interest were isolated using the QIAquick Gel Extraction Kit (Qiagen) according to the manufacturer's protocol and diluted to 1 ng/µl in DNase-/RNase-free water (Invitrogen), mixed with the respective forward or reverse primer (Table 2) and shipped to Eurofins Genomics for Sanger sequencing. Sequencing reads were analyzed using SnapGene Viewer (version 5, www.snapgene.com) and the UCSC Genome Browser[6,7].

Animal care

Animal experiments were approved by the authorities at Hannover Medical School and the Niedersächsische Landesamt für Verbraucherschutz und Lebensmittelsicherheit (LAVES) (animal experiment proposals 15/1978 and 17/143). Mice were randomly assigned to experimental groups and

strictly monitored according to the local regulations and animal experiment proposals for all experiments. TAC surgery was performed in 8-10 weeks old C57BL/6J and C57BL/6N mice (Charles River Laboratories) to induce a cardiac pressure-overload mouse model as previously described[8]. Experimental endpoints were at 8 and 12 weeks after TAC surgery at which either whole hearts were harvested or separated into different cell fractions via heart fractionation, respectively. All animal experiments were conducted in strict accordance with the Animal Research: Reporting of In Vivo Experiments (ARRIVE) guidelines, with concerted efforts to minimize the number of animals used. Sample size determination was based on empirical data from previous studies and was consistent with established practices in the field. Mice were assigned unique identifiers and randomly allocated to experimental or control groups using a standardized randomization protocol.

Organ harvest and fractionation of adult mouse hearts

Mice were anaesthetized with 2 % isoflurane in oxygen inside an inhalation chamber and euthanatized by cervical dislocation. Following disinfection, the thorax and the skull were opened to remove the following organs: aorta, brain, heart, kidney, liver, lung and spleen. Additionally, a biopsy of the skeletal muscle was collected from the upper leg. Tissue samples were cut into small pieces and snap frozen.

To evaluate expression of circRNAs within the major cell types of the failing heart, hearts of adult mice were fractionated 12 weeks after TAC surgery. Mice were fixed in the supine position on a hot plate and anesthetized with 2 % isoflurane in oxygen as previously described[8]. By inserting a blunt needle into the aorta, retrograde perfusion of the heart was performed with perfusion buffer (113 mM NaCl (Sigma), 4.7 mM KCl (Sigma), 600 µM KH_2PO_4 (Sigma), 600 µM Na_2HPO_4 Sigma) 1.2 mM $MgSO_4$-7·H_2O (Sigma), 32 µM phenol red (Sigma), 12 mM $NaHCO_3$ (Sigma), 10 mM $KHCO_3$ (Sigma), 10 mM HEPES (Sigma), 10 mM taurine (Sigma), 0.1 % glucose (Sigma) and 10 mM BDM (Sigma), pH 7.46) *in vivo* as well as ex vivo. Subsequently, hearts were perfused ex vivo with digestion buffer (perfusion buffer supplemented with 12.5 µM $CaCl_2$ (Sigma), 700 U/ml Collagenase type II (Worthington)) until the heart appeared swollen, pale and flaccid. The ventricles were separated from the atria, minced in digestion buffer and sheared before stopping the enzymatic digestion with perfusion buffer supplemented with 10 % FBS (Gibco) and 12.5 µM $CaCl_2$ (Sigma). Cells were sheared once more, filtered through a 100 µm MACS® Smart Strainer (Miltenyi Biotec), gently mixed with cardiac

fibroblast (CF) medium (MEM HBS with NEAA (Bioconcept), 4.2 mM $NaHCO_3$, 2 ng/ml Vitamin B12 (Sigma) and penicillin-streptomycin (100 U/ml and 100 µg/ml, Gibco)) and incubated for 10 min. The resulting supernatant was transferred into a new tube, while sedimented cardiomyocytes (CMs) were snap-frozen in liquid nitrogen. The supernatant was centrifuged and transferred into a new tube to separate CFs and endothelial cells (ECs) (supernatant) from the remaining CMs (pellet). CFs and ECs were then centrifuged, resuspended in CF medium, seeded onto a 10 cm dish (Sarstedt) and preplated. The resulting supernatant containing the ECs was transferred into a new tube and stored at 37°C while the attached CFs were thoroughly washed twice with DPBS and detached with a cell scraper (CytoOne) and snap frozen in liquid nitrogen. The EC fraction was centrifuged and the pellet resuspended in cold MACS® buffer (5 % MACS® BSA Stock Solution (Miltenyi Biotec) in autoMACS® Rinsing Solution (Miltenyi Biotec)). The cell suspension was mixed with CD146 (LSEC) MicroBeads (#130-092-007, Miltenyi Biotec) and incubated on ice. Following, the sample was washed with MACS® buffer, centrifuged and the resulting pellet resuspended in MACS® buffer. Next, the sample was loaded onto a prepared 30 µm pre-separation filter (Miltenyi Biotec) on top of a MS column (Miltenyi Biotec) and washed three times with MACS® buffer after the sample had passed through the column. Ultimately, ECs were eluted in MACS® buffer and snap-frozen in liquid nitrogen.

Isolation and purification of patient-derived cardiac fibroblasts

Supplementary Table 2: Non-HF HCFs

**Name**	**Non-HF #1**	**Non-HF #2**	**Non-HF #3**
Company	PromoCell	PromoCell	PromoCell
Cat. No.	C-12375	C-12375	C-12375
LOT No.	450Z014.1	452Z013.1	436Z024.3
Age	60 years	49 years	33 years
Sex	male	male	female
Ethnicity	caucasian	caucasian	caucasian

Supplementary Table 3: HF HCFs

**Name**	**HF #1**	**HF #2**	**HF #3**
Age	64 years	46 years	61 years
Sex	male	male	male
Disease	Dilated cardiomyopathy	Dilated cardiomyopathy	Ischemic cardiomyopathy
EF	10-15 %	13 %	13 %
Comorbidities	chronic kidney failure, hypothyreosis, atrial fibrillation, COVID-19	persistant atrial fibrillation, arterial hypertension, chronic kidney failure, hyperthyreiodism	3-vessel disease, arterial hypertension, diabetes mellitus type II, peripheral arterial disease

Non-HF HCFs were commercially purchased, while HF HCFs were isolated from human heart biopsies. Information about patients as well as their clinical conditions are listed in Supplementary Table 2 and Supplementary Table 3, respectively. The ethics committee of the Hannover Medical School permitted and approved isolation of HCFs from patient's biomaterial (number 9398_BO_K_2020). All patients were informed and gave consent prior to sample and information collection, and full written informed consent was obtained[9]. Biomaterial was collected from the KFO311 (MHH Register Herz-/Lungeninsuffizienz) during LVAD implantations and kindly provided by the pathology department. Biopsies were collected and stored in ice-cold HBSS (Gibco) on ice during the transport. The tissue was cut into pieces and digested (600 U/ml Collagenase II and 60 U/ml DNase I (AppliChem) in HBSS). Dissociation of the tissue was performed by pipetting up and down several times. The cloudy supernatant was removed and stored on ice, while the remaining non-dissociated tissue was incubated in fresh digestion buffer. Following, the cell suspension was filtered through a 100 µm MACS® Smart Strainer, washed once with HBSS and centrifuged. The supernatant was transferred to a fresh tube and centrifuged, while the CM-containing pellet was discarded. Next, the cell pellet was resuspended in FGM-3 and cultured until confluent. Ultimately HF HCFs were purified with anti-Fibroblast MicroBeads, human (#130-050-601, Miltenyi Biotec).

RNA stability assays

To evaluate resistance against exonuclease-mediated digestion, 1 µg of isolated, total RNA was digested with 3 U RNase R (Lucigen) for 30 min. For control samples, RNase R was replaced with PCR-grade water. Following digestion, RNA was reverse transcribed and analyzed via qRT-PCR as described earlier. RNA levels were normalized to control group and illustrated as percentage values.

Moreover, RNA stability was assessed by treating HCFs with 2 µg/ml actinomycin D (Sigma) for 0-24 h. RNA was isolated, reverse transcribed and analyzed via qRT-PCR as described earlier. RNA levels were normalized to 0 h.

Cloning of circIGF1R overexpression construct

Forward and reverse primers (Eurofins Genomics, Supplementary Table 4) were designed to bind within the flanking introns of the second exon of IGF1R, including a restriction site for ClaI and XbaI (underlined nucleotides indicate restriction enzyme sites), respectively. The Phusion® High-Fidelity PCR Master Mix (New England Biolabs) was utilized according to the manufacturer's protocol to amplify the circIGF1R-encoding exon from HCF gDNA. Genomic DNA was isolated via the DNeasy Blood & Tissue Kit (Qiagen) according to the manufacturer's protocol. The resulting amplicon was loaded on a 1 % agarose gel together with the Quick-Load® Purple 1 kb DNA Ladder (New England Biolabs) and extracted utilizing the QIAquick Gel Extraction Kit according to the manufacturer's protocol. The isolated PCR product was cloned into the pJET1.2/blunt Cloning Vector by utilizing the CloneJET™ PCR Cloning Kit (Thermo Fisher) according to the manufacturer's protocol.

Supplementary Table 4: Primer pairs for cloning the circIGF1R overexpression construct

**Primer**	**RE site**	**Sequence (5'→3')**
h-circIGF1R-cloning forward	ClaI	AATATCGATGTCTTGGGTATCTGCCAATAAT
h-circIGF1R-cloning reverse	XbaI	AATTCTAGAGGGCCAACACTTAAATAATAGC

The circIGF1R overexpression construct was generated via digestion with ClaI (New England Biolabs) and XbaI (New England Biolabs) and further sub-cloned into the overexpression plasmid pAAV-MCS

expression Vector (Cell Biolabs) according to the manufacturer's protocol. Sanger sequencing was used to validate insertion of the overexpression construct into the cloned plasmid.

Generation of recombinant circRNA mimics by in-vitro-transcription

The full circIGF1R-encoding exon was amplified from our overexpression plasmid using the Q5® High-Fidelity 2x Master Mix according to the manufacturer's protocol. Forward and reverse primer (Eurofins Genomics) were designed to bind both ends of the exon, whereas the forward primer included the T7 promotor sequence (underlined in Supplementary Table 5) as well as a "GGG" spacer motif. Upon PCR amplification, DNA was purified via the QIAquick PCR purification Kit according to the manufacturer's protocol and transcribed via IVT utilizing the RiboMAX™ Large Scale RNA Production Systems – T7 (Promega). Per reaction, 5x T7 Transcription Buffer, rATP (7.5 mM), rCTP (7.5 mM), rGMP (6.25 mM, Sigma), rGTP (1.25 mM) and rUTP (7.5 mM) were combined with the purified PCR product and incubated for 2 h at 37°C. Following, the DNA template was digested via DNase I (Qiagen). The *in vitro* RNA was precipitated with 3 M sodium acetate (pH 5.2, Sigma) and ice-cold isopropanol (Sigma) and the resulting RNA pellet was resuspended in PCR-grade water. To facilitate the circularization of the linear transcript, a DNA splint (Eurofins Genomics, Supplementary Table 5) complementary to the BSJ of circIGF1R was utilized (3:1 molar ratio between DNA splint and IVT RNA) and catalyzed by T4 DNA Ligase. We utilized the circularized transcript as circIGF1R mimics for overexpression, whereas leftover uncircularized transcript was utilized as linIGF1R mimic for comparison experiments. Final RNA purification of the circularized transcript (circIGF1R mimics) was performed as described earlier.

Supplementary Table 5: Oligonucleotides for synthesis and circularization of circRNA mimics

**Primer**	**Sequence (5'→3')**
h-circIGF1R-ivT forward	TAATACGACTCACTATAGGGTCTGCGGGCCAGGCATCGACAT
h-circIGF1R-ivT reverse	TTTTCTGGCAGCGGTTTGTG
h-circIGF1R-ivT splint	GGCCCGCAGATTTTCTGGCA

Circular conformation of circIGF1R mimics was validated via PCR (Hot StarTaq Master Mix Kit) with divergent primers (Table 2), followed by agarose gel electrophoresis and Sanger sequencing (Eurofins Genomics) as described earlier.

Moreover, the correct length of circIGF1R mimics was evaluated via denaturing agarose gel (1 % agarose and 0.06 % sodium hypochlorite (Carl Roth) in 1x TAE buffer (Carl Roth)) electrophoresis. RNA samples were loaded onto the polymerized gel and electrophoresis was performed in a SUB-CellR GT Cell electrophoresis chamber (Bio-Rad).

RNA fluorescence *in situ* hybridization

To detect circIGF1R localization in HCFs, RNA-FISH was performed with the ViewRNA™ Cell Plus Assay (Invitrogen) according to the manufacturer's protocol. Briefly, target probes were hybridized and fluorescence signal was amplified by a PreAmplifier as well as an Amplifier. A GAPDH probe was used as cytoplasmic control and nuclei were stained with DAPI. The stained cover slips were mounted on a glass slide (Klinipath) using Vectashield® Hard Set Mounting Medium (Vector Laboratories) and dried at 4°C in the dark. Images were captured on the following day using a CFI Plan Apo Lambda 60X Oil objective (Nikon) in a BZ-X810 inverted fluorescence microscope (Keyence) and processed with FIJI ImageJ (version 1)[10]. Probes utilized for RNA-FISH experiments (Invitrogen) are listed in Supplementary Table 6.

Supplementary Table 6: Probes for RNA-FISH experiments

**Target**	**Type**	**Target Sequence (5'→3') / Cat. No.**
h-circIGF1R	1	GCTGCTGGACCACAAACCGCTGCCAGAAAATCTGCGGGCCAGGC ATCGACATCCGCAACG
h-GAPDH	4	VX-06

Subcellular fractionation

HCFs were lysed in cell lysis buffer (10 mM Tris-HCl (pH 8, Carl Roth), 140 mM NaCl (Carl Roth), 1.5 mM $MgCl_2$ (Carl Roth), 0.5 % IGEPAL® CA-630 (Sigma), 10 % cOmplete™ Protease Inhibitor Cocktail (Roche)) and centrifuged. The resulting supernatant containing the cytoplasmic fraction was collected, whereas the pellet was washed twice with cell lysis buffer, resuspended in nuclear lysis buffer (10 mM Tris-HCl (pH 8), 140 mM NaCl, 1.5 mM $MgCl_2$, 0.5 % IGEPAL® CA-630, 1 % Tween® 20 (Carl Roth), 10 % cOmplete™ Protease Inhibitor Cocktail) and centrifuged. The pellet containing the nuclear fraction was collected in nuclear lysis buffer.

WST-1 assay

HCFs were transfected with either siRNAs or mimics. After 48 h, the transfected cells were incubated with 10 % Cell Proliferation Reagent WST-1 (Roche). Absorbance was measured at 450 nm and 630 nm (reference wavelength) using a Cytation 1 Cell Imaging Multimode Reader (BioTek). Wells without any cells and with FGM-3 containing WST-1 served as a background control. For the analysis, the mean absorbance of the background control wells was subtracted from each well and the absorbance at 630 nm was subtracted from the absorbance at 450 nm. Each biological replicate consists of four technical replicates.

Carboxyfluorescein succinimidyl ester flow cytometry

HCFs were treated with 100 ng/ml Nocodazole (Merck) for 20 h to synchronize the cells. Next, HCFs were incubated with 5 µM CellTrace™ CFSE (Invitrogen) for 20 min in the dark. Cells were seeded following centrifugation and washing to remove unbound dye. The subsequent day, HCFs were transfected with either siRNAs or mimics. The cells were harvested 72 h after transfection and fluorescence intensity was measured with a Cytoflex S (Beckman Coulter) using a 488 nm laser as well as a FITC bandpass filter (λ = 525/40 nm) to evaluate proliferation. Dead cells as well as cell duplicates were excluded as part of the gating strategy and signal intensity was adjusted using an unstained cell sample. For the analysis, a threshold in the FITC channel was adjusted so that 50 % of the control cells exhibited a CFSE signal below this threshold and globally applied. Proliferation was quantified by assessing the amount of cells in each group below the globally set threshold. Each biological replicate consists of three technical replicates.

5-bromo-2'-deoxyuridine ELISA

The Cell Proliferation ELISA, BrdU (colorimetric) (Roche) was utilized to quantify proliferation of HCFs. HCFs were cultured in FGM-3 containing 0.1 % 5-bromo-2'-deoxyuridine (BrdU) 48 h after transfection with siRNAs or mimics. Following 16 h of incubation, the assay was conducted according to the manufacturer's protocol. Absorbance was measured at 370 nm and 490 nm (reference wavelength) in a Synergy HT Microplate Reader (BioTek). FGM-3 containing 0.1 % BrdU inside wells without any cells served as a background control. For the analysis, the mean absorbance of the

background samples was subtracted from each sample and absorbance at 490 nm was subtracted from the absorbance at 370 nm. Each biological replicate consists of six to eight technical replicates.

Immunocytochemistry

HCFs were transfected with siRNAs or mimics and incubated for 48 h. The transfected cells were fixated in 4 % PFA (Carl Roth) in PBS, followed by permeabilization with 0.1 % Triton® X 100 (Carl Roth) in PBS. Following blocking of unspecific binding sites with 5 % donkey serum (Bio-Rad) in PBS, cells were incubated with anti-KI67 antibody (#ab16667, 1:250, Abcam) overnight at 4°C. On the following day, cells were washed three times with PBS and incubated with anti-rabbit IgG, AF488-conjugated (1:500, Invitrogen) as well as 5 µg/ml Hoechst 33342 (Invitrogen). Ultimately, cells were washed three times PBS before adding PBS to prevent drying. Cells were imaged in a Cytation 1 Cell Imaging Multimode Reader (BioTek) using a 20x Olympus Plan Fluorite Phase Objective (NA: 0.45; Agilent) and a DAPI (λ_{Ex} = 377/50 nm, λ_{Em} = 447/60 nm; Agilent) as well as a GFP (λ_{Ex} = 469/35 nm, λ_{Em} = 525/39 nm; Agilent) filter cube. For each sample, 25 different spots within the well were imaged to capture a representative amount of cells. Gen5 (version 3, BioTek) and FIJI ImageJ (version 1)[10] were used to process and analyze the images. To analyze proliferation, the number of $KI67^+$ nuclei was divided by the number of total nuclei counted and depicted in percent. Each biological replicate consists of three to four technical replicates.

Inhibition of glycolysis via 2-deoxyglucose

At 24 h after transfection of siRNAs, HCFs were treated with 5 mM 2-DG (Carl Roth) in FGM-3 for 24 h. Control cells were treated with the same volume of sterile ddH_2O (vehicle control).

Real-time metabolic flux analysis

Glycolytic activity was assessed utilizing the Seahorse XF Mito Stress Test Kit (Agilent) according to the manufacturer's protocol. HCFs were transfected with siRNAs or mimics for 24 h. Cells were washed and cultured in Seahorse Assay Buffer: Seahorse XF DMEM medium, pH 7.4 (Agilent) supplemented with 10 mM glucose (Agilent), 1 mM pyruvate (Agilent) and 2 mM L-glutamine (Sigma). Rotenone/antimycin A (5 µM) and 2-DG (500 mM) were diluted in Seahorse Assay Buffer before loading them onto the sensor cartridge. Data was collected by a Seahorse XFe96 Analyzer (Agilent).

Upon completion of the data reads, Hoechst 33342 (3.33 µg/ml) was added to stain nuclei. Ultimately, cells were imaged in a Cytation 1 Cell Imaging Multimode Reader (BioTek) using a 4x Olympus Plan Fluorite Objective (NA: 0.13; Agilent) and a DAPI (λ_{Ex} = 377/50 nm, λ_{Em} = 447/60 nm; Agilent) filter cube. Nuclei were counted using Gen5 (version 3, BioTek) and data readouts were normalized to the cell count using Seahorse Wave Desktop (version 2, Agilent). Each biological replicate consists of six to twelve technical replicates.

Glucose and lactate measurement from cellular supernatants

To evaluate glucose uptake and lactate secretion of HCFs, cellular supernatant was collected 24 h after transfection with siRNAs or mimics. Concentration of glucose and lactate in supernatants were measured utilizing a 2950D Biochemistry Analyzer (YSI) and quantified via measuring respective standards as described[11]. Each biological replicate consists of three technical replicates.

Tracing of intracellular glucose trafficking

Following the transfection with siRNAs or mimics, HCFs were cultivated in FGM-3 tracer medium: Customer Formulation FBM-3 (PromoCell), without glucose and L-glutamin, supplemented with FGM-3 SupplementMix (PromoCell), penicillin-streptomycin (100 U/ml and 100 µg/ml, Gibco), 8 mM [U-$^{13}C_6$]glucose (Cambridge Isotope Laboratories) and 5 mM L-glutamine (Sigma) for 24 h. On the day of harvesting, cells were washed once with 0.9 % NaCl (Carl Roth) before adding ice-cold methanol (Carl Roth) as well as ice-cold ddH_2O. HCFs were detached with a cell scraper and transferred into a tube containing ice-cold chloroform (Carl Roth), followed by incubation for 20 min at 1400 rpm and 4°C. Phases were separated via centrifugation and the upper aqueous phase was collected. Precedent to isotopic measurement, samples were transferred into a conically shaped glass vial and dried in a CentriVap Vacuum Concentrator (Labconco) overnight. Following, dried metabolite extracts were derivatized with 20 mg/ml methoxyamine-hydrochloride (Sigma) in pyridine (Carl Roth) as well as MSTFA (Chromatographie-Service) or MTBSTFA (Restek) and measured in full scan mode or selected ion mode[11,12]. Isotopic enrichment was measured via GC/MS in a 7890B gas chromatograph (Agilent) equipped with a 30-m DB-35ms and a 5-m DuraGuard capillary column (Agilent) for separation of derivatized metabolites, and a 5977B MSD system (Agilent) for measurement of metabolites[11].

Processing chromatograms and calculating mass isotopomer distributions was performed by Metabolite Detector Software[11,13]. Each biological replicate consists of three technical replicates.

RNA Pulldown

RNA pulldown experiments were performed as previously described[14]. In brief, 10^7 HCFs were washed and resuspended in cell lysis buffer (50 mM Tris pH 7 (Roth), 10 mM EDTA (Invitrogen), 0.1 % SDS (Roth), 1 mM DTT (Sigma), 100 U/ml RNasin® Ribonuclease Inhibitor (Promega), cOmplete™ Protease Inhibitor (Roche)) and broken down mechanically. Insoluble cellular components were removed by centrifugation and 5 % of the cleared lysate were as input control. The remaining lysate was mixed with two volumes of hybridization buffer (0.5 M NaCl (Sigma), 0.1 % SDS, 50 mM Tris pH 7, 1 mM EDTA, 15 % formamide (Ambion), 1 mM DTT, 100 U/ml RNasin® Ribonuclease Inhibitor, cOmplete™ Protease Inhibitor) and incubated with 10 µg biotynilated DNA probe (Supplementary Table 7) for 3 h. In the meantime, 100 µl Dynabeads™ MyOne™ Streptavidin C1 (Invitrogen) were washed thrice with bead wash buffer (5 mM Tris-HCl pH 7.5 (Roth), 0.5 mM EDTA, 1 M NaCl), twice with solution A (100 mM NaOH (Sigma), 50 mM NaCl) and once in solution B (100 mM NaCl). Following, the washed beads were blocked (1 µg/µl BSA (Ambion), 1 µg/µl Yeast tRNA (Invitrogen)) for 2 h at 4°C. Blocked beads were washed twice with lysis buffer, resuspended in lysis buffer and incubated with the cell lysate for 1 h. Subsequently, samples were washed five times with wash buffer (10 mM Tris-HCl pH 7.5, 10 mM KCl (Roth), 1.5 mM MgCl2 (Merck), 150 mM NaCl, 5 mM DTT, 60 U/ml RNasin® Ribonuclease Inhibitor, cOmplete™ Protease Inhibitor) and three times with MS Buffer (10 mM Tris-HCl pH 7.5, 150 mM NaCl). Washed samples were divided into RNA (20 %) and protein (80 %).

Supplementary Table 7: Probes for RNA pulldown experiments

**Probe**	**Modification**	**Sequence (5’→3’)**
NC	5’Biosg	AAAAGGTAGTGTAATCGCCTTGTT
h-circIGF1R	5’Biosg	AAACGATGCCTGGCCCGCAGATTTTCTGGCAGC

Mass spectrometry analysis

Preparation of protein samples and mass spectrometry experiments were conducted as previously described[14]. RNA pulldown samples were alkylated with 4 % acrylamide (Applichem) and separated on a 4-15 % Mini-PROTEAN® TGX™ Precast Protein Gel (Bio-Rad). Coomassie Brilliant Blue G-250 (Thermo Fisher) was utilized to stain proteins and washing twice with ddH_2O destained the gel. Lanes were cut into four pieces and digested within the gel using trypsin. Digested peptides were extracted and analyzed utilising an Orbitrap MS (Thermo Fisher). Procession of raw data was performed using MaxQuant[15] as well as Perseus[16] and searched against reviewed human entries of uniprot database and identification of proteins was controlled by a FDR ≤ 0.01 on protein and peptide level. Protein intensities were normalized to median values to correct for varying protein loads. Additionally, only proteins, which were identified in all biological replicates, were considered for quantification. Enriched proteins were identified by normalising to NC probe as well as input to identify putative binding partners. *In silico* interaction prediction of circIGF1R against the RNA-binding proteome was performed with catRAPID (http://s.tartaglialab.com/page/catrapid_group).

Western Blot

For electrophoretic band separation, protein fractions from pulldown experiments were reconstituted in ddH_2O and mixed with protein loading dye (NEB), incubated at 95°C for 5 minutes and loaded onto 12 % SDS gels together with Precision Plus Protein™ WesternC™ Blotting Standards (Bio-Rad) and run at 30 V. Following, proteins were blotted on a PVDF membrane (Bio-Rad) using a Mini Trans-Blot® Cell (Bio-Rad) system at 4°C overnight. The membrane was blocked in 5 % milk (Carl Roth) in TBST before incubation with primary antibodies for AZGP1 (Proteintech) and Vinculin (Sigma) diluted in TBST (1:1000 and 1:2500, respectively) at 4°C overnight. HRP-linked secondary antibodies (Cell Signaling) were added after washing with TBST and incubated for 1Clarity Western ECL Substrate (Bio-Rad) and imaged using a ChemiDoc MP Imaging System (Bio-Rad).

References

1 Sievers, F. *et al.* Fast, scalable generation of high-quality protein multiple sequence alignments using Clustal Omega. *Mol Syst Biol* **7**, 539 (2011). https://doi.org:10.1038/msb.2011.75

2 Larkin, M. A. *et al.* Clustal W and Clustal X version 2.0. *Bioinformatics* **23**, 2947-2948 (2007). https://doi.org:10.1093/bioinformatics/btm404

3 Thum, T. & Borlak, J. Gene expression in distinct regions of the heart. *Lancet* **355**, 979-983 (2000). https://doi.org:10.1016/S0140-6736(00)99016-0

4 Viereck, J. *et al.* Targeting muscle-enriched long non-coding RNA H19 reverses pathological cardiac hypertrophy. *Eur Heart J* **41**, 3462-3474 (2020). https://doi.org:10.1093/eurheartj/ehaa519

5 Lu, D. *et al.* A circular RNA derived from the insulin receptor locus protects against doxorubicin-induced cardiotoxicity. *Eur Heart J* **43**, 4496-4511 (2022). https://doi.org:10.1093/eurheartj/ehac337

6 Kent, W. J. BLAT--the BLAST-like alignment tool. *Genome Res* **12**, 656-664 (2002). https://doi.org:10.1101/gr.229202

7 Kent, W. J. *et al.* The human genome browser at UCSC. *Genome Res* **12**, 996-1006 (2002). https://doi.org:10.1101/gr.229102

8 Piccoli, M. T. *et al.* Inhibition of the Cardiac Fibroblast-Enriched lncRNA Meg3 Prevents Cardiac Fibrosis and Diastolic Dysfunction. *Circ Res* **121**, 575-583 (2017). https://doi.org:10.1161/CIRCRESAHA.117.310624

9 Schmidt, K. *et al.* SGLT2 inhibitors attenuate endothelial to mesenchymal transition and cardiac fibroblast activation. *Sci Rep* **14**, 16459 (2024). https://doi.org:10.1038/s41598-024-65410-9

10 Schindelin, J. *et al.* Fiji: an open-source platform for biological-image analysis. *Nat Methods* **9**, 676-682 (2012). https://doi.org:10.1038/nmeth.2019

11 He, W. *et al.* Mesaconate is synthesized from itaconate and exerts immunomodulatory effects in macrophages. *Nat Metab* **4**, 524-533 (2022). https://doi.org:10.1038/s42255-022-00565-1

12 Battello, N. *et al.* The role of HIF-1 in oncostatin M-dependent metabolic reprogramming of hepatic cells. *Cancer Metab* **4**, 3 (2016). https://doi.org:10.1186/s40170-016-0141-0

13 Hiller, K. *et al.* MetaboliteDetector: comprehensive analysis tool for targeted and nontargeted GC/MS based metabolome analysis. *Anal Chem* **81**, 3429-3439 (2009). https://doi.org:10.1021/ac802689c

14 Neufeldt, D. *et al.* Circular RNA circZFPM2 regulates cardiomyocyte hypertrophy and survival. *Basic Res Cardiol* **119**, 613-632 (2024). https://doi.org:10.1007/s00395-024-01048-y

15 Cox, J. & Mann, M. MaxQuant enables high peptide identification rates, individualized p.p.b.-range mass accuracies and proteome-wide protein quantification. *Nat Biotechnol* **26**, 1367-1372 (2008). https://doi.org:10.1038/nbt.1511

16 Cox, J. & Mann, M. 1D and 2D annotation enrichment: a statistical method integrating quantitative proteomics with complementary high-throughput data. *BMC Bioinformatics* **13 Suppl 16**, S12 (2012). https://doi.org:10.1186/1471-2105-13-S16-S12
