# Supplementary material for: Circular RNA circIGF1R controls cardiac fibroblast proliferation through regulation of carbohydrate metabolism
Source: Sci Rep. 2025 Jun 27;15:20331. doi: 10.1038/s41598-025-07167-3 (PMC12205068; doi:10.1038/s41598-025-07167-3)
Supplement: Supplementary file 2 — Supplementary Material 2 [file 41598_2025_7167_MOESM2_ESM.pptx]

## Slide 1
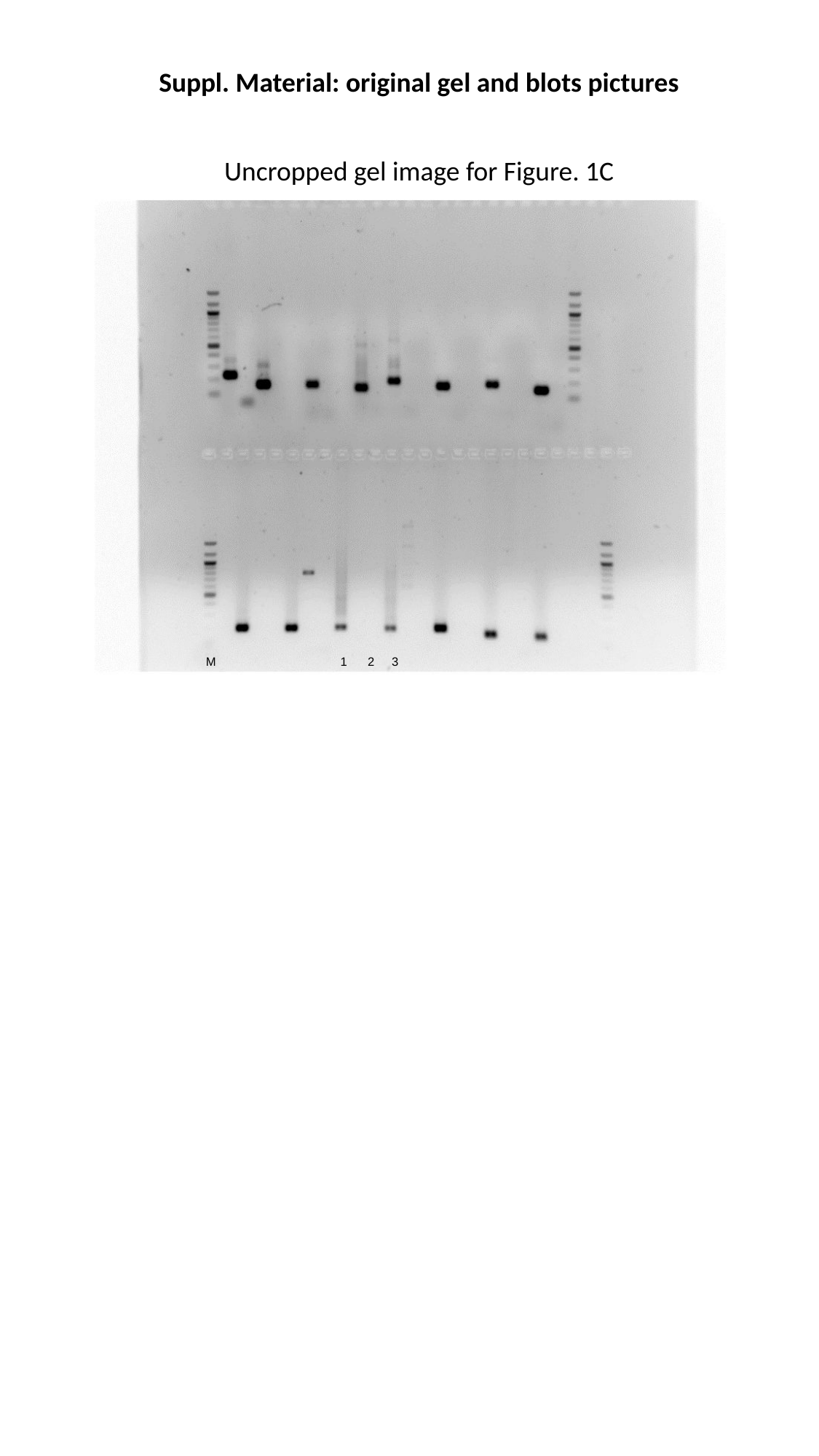

Suppl. Material: original gel and blots pictures
Uncropped gel image for Figure. 1C
M 1 2 3

## Slide 2
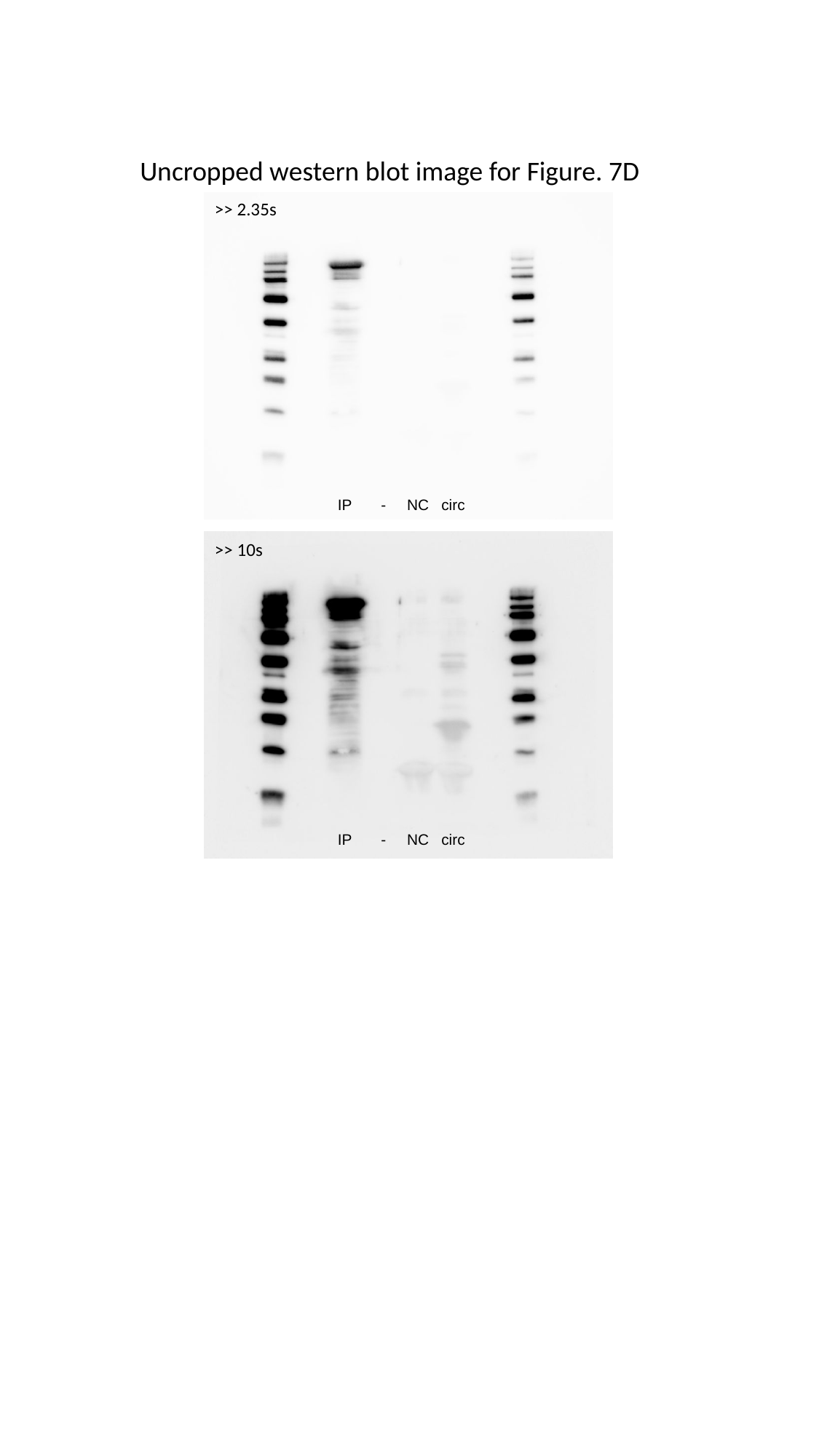

Uncropped western blot image for Figure. 7D
IP - NC circ
>> 2.35s
IP - NC circ
>> 10s

## Slide 3
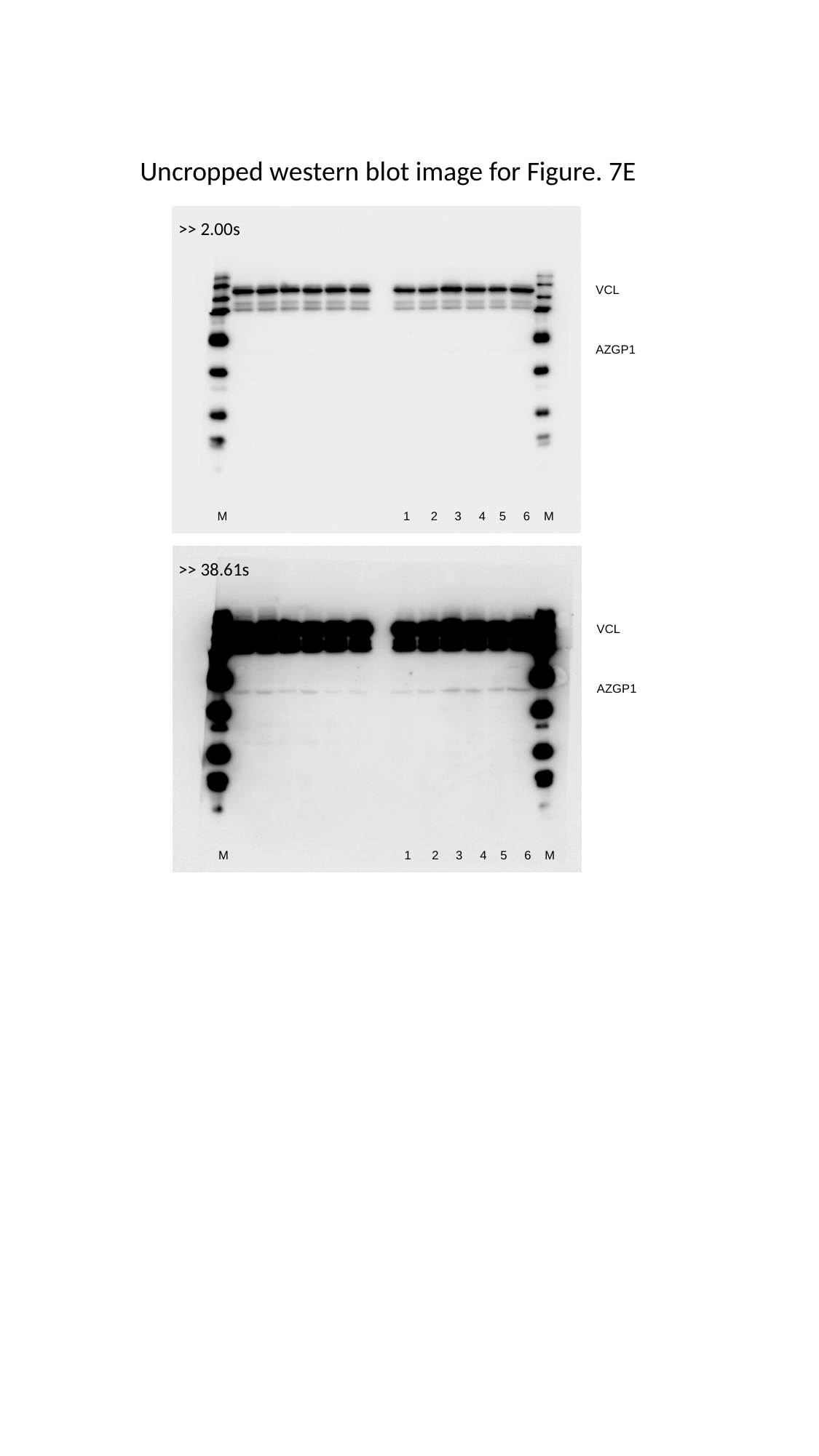

Uncropped western blot image for Figure. 7E
VCL
AZGP1
M 1 2 3 4 5 6 M
>> 2.00s
VCL
AZGP1
M 1 2 3 4 5 6 M
>> 38.61s

## Slide 4
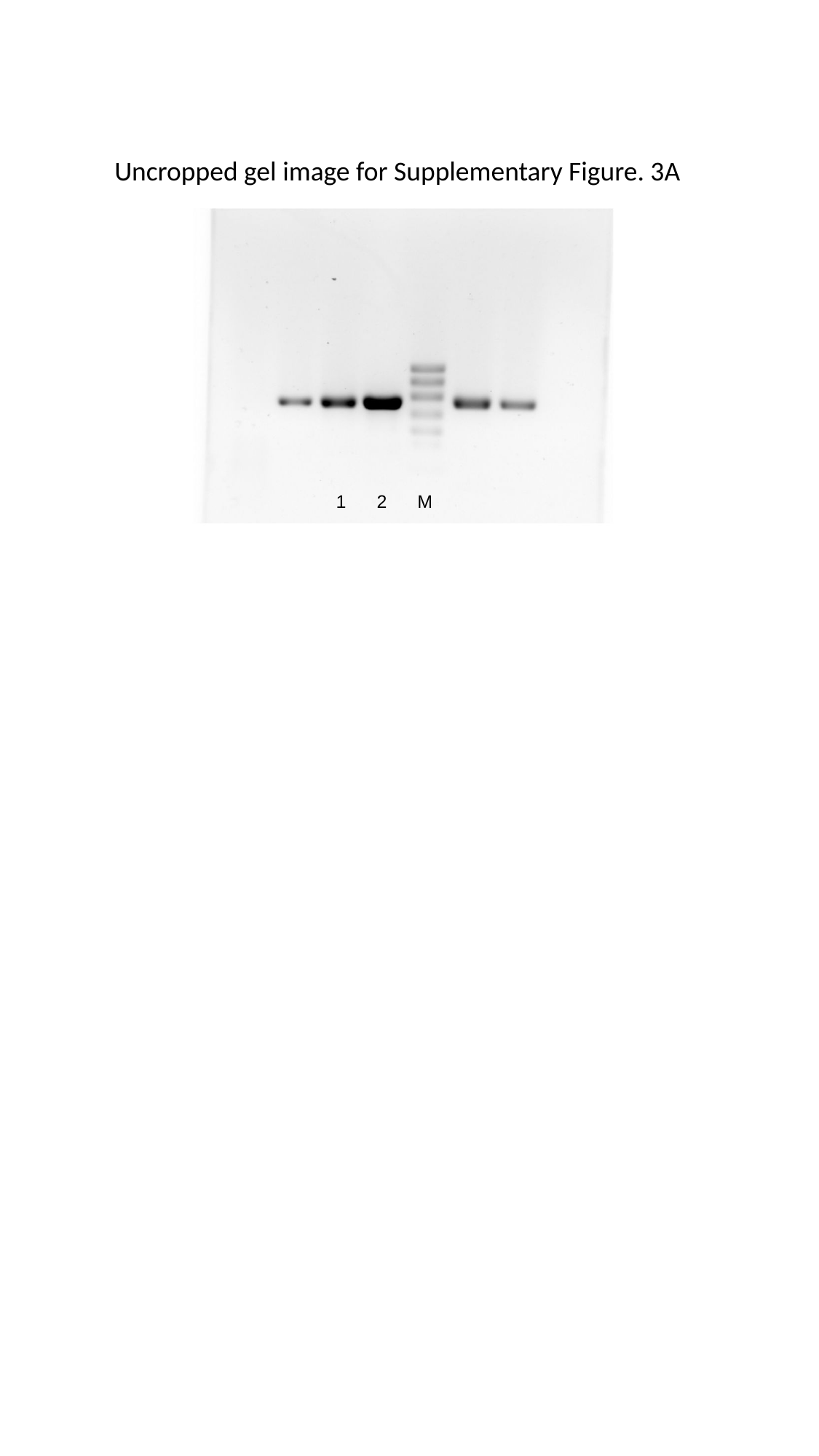

Uncropped gel image for Supplementary Figure. 3A
1 2 M

## Slide 5
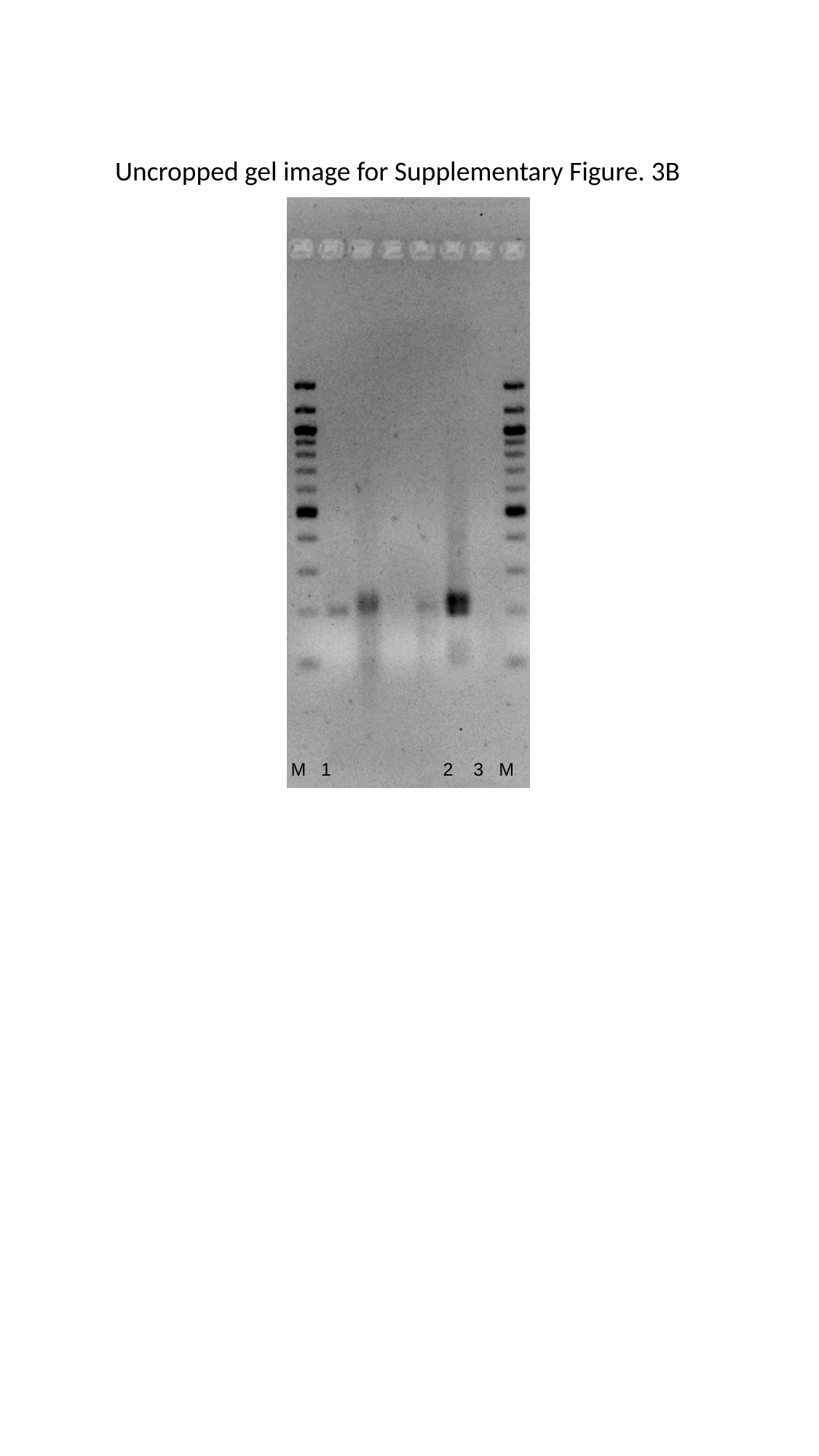

Uncropped gel image for Supplementary Figure. 3B
M 1 2 3 M
